# Assessment of the acute toxicity of ethanol extract from the rhizomes of *Iris scariosa* L. in mice

**DOI:** 10.3389/fphar.2025.1701980

**Published:** 2026-01-12

**Authors:** Ailazzat A. Aitkenova, Gayane A. Atazhanova, Karakoz Zh. Badekova, Aleksandr V. Samorodov, Saule B. Akhmetova, Margarita Yu. Ishmuratova, Islambek Karilkhan, Arailym A. Tazhina, Ainur Zh. Sydykova

**Affiliations:** 1 School of Pharmacy, Karaganda Medical University, Karaganda, Kazakhstan; 2 V.A. Negovsky Scientific Research Institute of General Reanimatology of the Federal Research and Clinical Center of Intensive Care Medicine and Rehabilitology, Moscow, Russia; 3 Department of Biomedicine, Karaganda Medical University, Karaganda, Kazakhstan; 4 Research Park of Biotechnology and Eco-Monitoring, Karaganda Buketov University, Karaganda, Kazakhstan; 5 Department of Informatics and Biostatistics, Karaganda Medical University, Karaganda, Kazakhstan

**Keywords:** *I. scariosa* L., acute toxicity, ethanol extract, laboratory mice, histopathology, phytotherapy

## Abstract

**Background:**

Plants of the genus *Iris* L. (Iridaceae) attract the attention of researchers as a source of biologically active phenolic compounds with pronounced antioxidant, anti-inflammatory, and cytoprotective properties. However, the toxicological assessment of individual species, including *Iris scariosa* L., remains limited, which hinders their pharmaceutical development.

**Aim:**

The aim of this study was to evaluate the acute oral toxicity of ultrasonic ethanol extract of *I. scariosa* rhizomes in laboratory mice in accordance with OECD Guideline 425 (2001) and the regulatory requirements of the Ministry of Health of the Republic of Kazakhstan.

**Methods:**

The extract was administered once intragastrically at doses of 500, 1000, and 2000 mg/kg, followed by 14 days of observation of clinical status, body weight dynamics, hematological, biochemical, and histomorphological parameters.

**Results:**

No fatalities were recorded. Blood parameters remained within physiological norms (p > 0.05). At the maximum dose, a slight, statistically insignificant increase in ALT and AST activity was observed, reflecting a compensatory response of hepatocytes without signs of cytolysis. Histopathological analysis revealed moderate reversible changes in renal tissue, characterized by vacuolization of the tubular epithelium, venous hyperemia, and mild inflammatory infiltration, while maintaining normal glomerular structure and absence of fibrosis. The morphology of the liver, pancreas, heart, and spleen remained unchanged.

**Conclusion:**

According to the OECD/GHS classification, *I. scariosa* extract is classified as a Category V substance (LD_50_ > 2000 mg/kg), which indicates low acute oral toxicity and good tolerability. The morphological reactions observed are temporary and adaptive in nature, probably associated with the biphasic response of phenolic compounds. The results confirm the safety of the extract and the need for further study of its chronic toxicity.

## Introduction

Medicinal plants remain an important source of biologically active compounds with proven therapeutic potential (Sall et al., 2025; Atazhanova et al., 2022). According to the World Health Organization, herbal medicines are still widely used due to their accessibility and safety (World Health Organization, 2013). Modern herbal medicine combines traditional knowledge and pharmacological research aimed at finding natural substances with proven efficacy and low toxicity (Tine et al., 2024; Levaya et al., 2025; Itzhanova et al., 2024; Zholdasbayev et al., 2023).

The genus *Iris Tourn.* exL. (family Iridaceae) comprises about 400 species, distributed mainly within the Holarctic region of the northern hemisphere, with the exception of the Arctic regions (Plant of the World Online, 2025). Of these, 21 species are registered in Kazakhstan (Flora of Kazakhstan, 1958). These plants are known for their rich phytochemical composition, including flavonoids, isoflavones, phenolic acids, triterpenoids, and aromatic compounds (Yousefsani et al., 2021; Bogdanov et al., 2022; Aitkenova et al., 2025a). Plants of this genus attract the attention of pharmacologists due to their wide range of proven biological activity, including antioxidant (Khatib et al., 2022), antimicrobial (Okba et al., 2022), antibacterial (Hoang et al., 2020), anti-inflammatory, anti-allergic, cytotoxic, and antiviral (including against coronavirus 229E) effects (Mykhailenko et al., 2020; Morozova et al., 2022).

In traditional medicine, species of the genus *Iris* L. are widely used in various regions of the world. In India, rhizomes are used as a diuretic, astringent, and laxative; in European countries, as a diaphoretic and expectorant; in Russia, in the form of decoctions for inflammatory diseases of the respiratory tract, such as bronchitis (Tikhomirova et al., 2015; Alekseeva et al., 2023). Folk sources particularly highlight plants with purple flowers, which are believed to have more pronounced therapeutic activity, probably due to the variability in the composition of essential oils and the concentration of bioactive components (Amin et al., 2017). Modern research confirms the pharmacological significance of individual components and extracts of Iris L. plants, which demonstrate antimicrobial activity against a wide range of pathogens (Aitkenova et al., 2025b; Chikhi et al., 2012; Ghasemi et al., 2025). Thus, the combination of traditional knowledge and modern scientific data confirms the promise of further study of representatives of the genus *Iris* L. as sources of biologically active compounds.

*Iris scariosa* Willd. ex Link is one of the species growing in Central Asia, including Kazakhstan. The underground parts of the plant contain a wide range of phenolic compounds, including flavonoids, isoflavones, benzophenones, and fatty acids. The petroleum ether fraction is characterized by a high content of saturated fatty acids and aromatic terpenoids (α-iron, β-ionone) (Omarova et al., 2024). The ethanol extract is rich in flavonoids—quercetin, rutin, apigenin, and artemisinin. Isoflavones (irilon, iriflogenin, tectorigenin) and compounds new to the Iridaceae family—artemisinin and pinoembrin—have been identified in the tert-butylmethyl fraction. These results are consistent with the literature data on the species *I. germanica* and *I. pseudacorus*, which have been shown to have pronounced antioxidant and anti-inflammatory properties (Bakhoda et al., 2025), but the toxicological characteristics of the species *I. scariosa*, which grows in Kazakhstan, have hardly been studied. Available data on other species of the genus Iris indicate their good tolerability and absence of pronounced toxicity (Jianv et al., 2018). *I. versicolor* L. root extract is recommended in a daily dose of 400–2400 mg and is considered safe for therapeutic use (Khatib et al., 2022). Similar studies of dry hydrophilic extracts of *I. pseudacorus* showed no toxic effects on laboratory animals (Khokhlova et al., 2012; Maru et al., 2025; Somarathna et al., 2021). The aim of this study was to evaluate the acute toxicity of ethanol extract from the rhizomes of *I. scariosa* L. in mice with analysis of morphological and biochemical changes.

## Materials and methods

### Extraction conditions and chemical characteristics

The object of the study was the underground organs of *Iris scariosa* Willd. ex Link., collected during the flowering phase in the Karaagash Forestry (Zhanaarkinsky District, Ulytau Region, Kazakhstan), located at an altitude of about 330–350 m above sea level (48.889173, 70.907358). The plant material was harvested between April and May 2024. The identification was carried out at the Department of Botany at the E.A. Buketov Karaganda University. The herbarium specimen is deposited in the Department’s Herbarium under accession number QAR12220.

Ultrasonic extraction was used to extract biologically active substances from the underground organs of *I. scariosa*. A sample of dried raw material (10.0 g) was poured with 300 ml of solvent (distilled water or ethanol:water mixtures) in a ratio of 1:10 (volume/volume). Extraction was carried out using an Ultrasonic Cleaner Sonic-3 device at a frequency of 40 kHz, a temperature of 20–22 °C, and a processing time of 30 minutes. The combined solutions were filtered through a paper filter and evaporated on a rotary evaporator at 50 °C, then the residual solvent was removed in a water bath at 60 °C. The resulting thick extracts were dark brown masses with a specific odor; the dry residue yield was 18.7% (w/w) of the mass of the starting material. The analysis of phenolic compounds in ultrasonic extracts of *I. scariosa* was performed using high-performance liquid chromatography coupled with ultraviolet detection and real-time tandem mass spectrometry (HPLC-UV-ESI-MS). Acetonitrile (ACN) for HPLC (≥99.9%, Sigma-Aldrich, France), formic acid (99–100%, AnalaR NORMAPUR®, VWR Chemicals, France), and high-purity water obtained using a Milli-Q system (Millipore, France) were used for the analysis. Twenty phenolic compounds were used as standards: catechin, epicatechin, naringenin, rutin, luteolin-7-O-glucoside, quercetin-3-glucoside, dihydroquercetin, myricetin, quercetin, naringenin, apigenin, luteolin, kaempferol, caffeic acid, gallic acid, chlorogenic acid, ferulic acid, p-coumaric acid, rosmarinic acid, cinnamic acid (Sigma-Aldrich, United States). Standards and samples were dissolved in a mixture of acetonitrile : water = 1:1 (v/v) and filtered through 0.22 μm membranes (PTFE). Chromatography was performed on an Agilent 1260 Infinity HPLC system (Agilent Technologies, USA) equipped with a G1311C 1260 Pump VL four-channel pump, a G1329B 1260 ALS autosampler, a G1316A 1260 TCC column thermostat, a G1314C 1260 VWD VL UV detector, and a G6130A LC-MS mass spectrometer with an ESI ionization source. System control and data processing were performed in the ChemStation B.04.03 SP1 software environment (Agilent Technologies). Separation was performed on a Zorbax Eclipse Plus C18 column (150 × 4.6 mm, 3.5 µm; Agilent Technologies, USA) at a temperature of 30 °C. The mobile phase consisted of phase A—water with 2.5% formic acid and phase B—acetonitrile with 2.5% formic acid. Elution was performed in gradient mode (Table 1). The flow rate was 0.4 mL/min, and the injection volume was 20 µL. The UV detection wavelengths were 280 and 360 nm (Ivasenko et al., 2021).

**TABLE 1:** Gradient Elution Program (A—water + 2.5% formic acid; b—acetonitrile + 2.5% formic acid)

Time (min)	%B	%A
0.00	3	97
7.00	20	80
7.10	30	70
27.00	40	60
35.00	50	50
36.00	20	70
40.00	3	97
50.00	3	97

### Laboratory animals and conditions of maintenance

Clinically healthy white outbred mice of both sexes (♂ and ♀) weighing 27–39 g were used to assess acute toxicity. All animals were kept in the vivarium of the Karaganda Medical University in conditions that met the standards of bioethics and the European Convention for the Protection of Vertebrate Animals Used for Scientific Purposes, as well as in accordance with the domestic “Guidelines for the Experimental (Preclinical) Study of New Pharmacological Substances” (Jegnie et al., 2023; Council of Europe, 1986; Khabriev, 2005).

The mice were housed in individual plastic cages with sterilized wood shavings bedding and had constant access to water and food. Optimal microclimate parameters were maintained: air temperature 21 °C ± 2 °C, relative humidity 50% ± 10%, natural ventilation, and light regime (12-h “day-night” cycle). A 14-day acclimatization period was conducted before the start of the experiment.

### Preparation of the extract and calculation of the dosage

A thick ultrasonic extract of *I. scariosa* L., obtained using 70% ethanol, was used as the test substance. Immediately before administration, the preparation was weighed and dissolved in distilled water to prepare a suspension. The volume of administration was 0.2 mL per animal, taking into account individual body weight. The extract was administered once, intragastrically, using a gastric tube. The animals were not fed for 2–3 h before and after administration. The control group was administered an equivalent volume of distilled water.

### Study design and acute toxicity assessment

The dose range (500–2000 mg/kg) was selected based on literature data and OECD Test Guideline 425 (Organisation for Economic Co-operation and Development (OECD), 2001) recommendations, according to which 2000 mg/kg is the maximum dose for testing substances with presumed low toxicity. The upper limit was taken as the limit dose that does not require further increase, and the lower levels (500 and 1000 mg/kg) were used to assess possible dose-dependent effects. Additionally, the choice of range is confirmed by experimental data for closely related species of the genus Iris, such as *I. versicolor* L. and *I. germanica* L., whose extracts did not cause acute toxicity at doses up to 2400 mg/kg (Khatib et al., 2022). Thus, the selected doses allow for the study of possible subtoxic changes while maintaining animal safety and comply with international regulatory requirements for preclinical studies of plant extracts.

The animals were divided into four groups of 8 individuals of both sexes in each. The distribution and dosage scheme is presented in Table 2.

**TABLE 2:** Experimental allocation and dosage regimen of *I. scariosa* L. in sex-specific mouse groups for acute toxicity study.

Group name	Gender (M/F)	Number of animals (n)	Administered substance	Dose (mg/kg)	Administration volume (mL)
A	M	4	*I. scariosa* L. ultrasonic extract in 70% ethanol	500	0.2
	F	4			
B	M	4		1,000	0.2
	F	4			
C	M	4		2000	0.2
	F	4			
D (control)	M	4		Distilled water	1.0

Each group received a single intragastric administration of the appropriate dose. The animals were monitored daily for 14 days. The following parameters were assessed: general clinical condition, appearance, motor activity, behavior, response to tactile and auditory stimuli, feed and water consumption, urination and defecation functions. Possible pathological signs were monitored: tremors, convulsions, muscle rigidity, coordination disorders, pathological postures, signs of self-injury, as well as manifestations of Straube syndrome (Belozertseva et al., 2015).

Additional indicators such as respiration, sedation, diarrhea, coat condition, skin color, and loss of consciousness were also monitored. Body weight was recorded at baseline and on days 1, 5, 10, and 14 of the experiment.

### Assessment of hematological and biochemical indicators

In order to study the possible toxic effects of *I. scariosa* L. extract, studies of hematological and biochemical blood parameters were conducted in white laboratory mice (Oecd, 2025). Before blood sampling, the animals were fasted for 18 h with free access to drinking water. Blood for hematological analysis was collected from the tail vein into vacuum tubes containing ethylenediaminetetraacetic acid (EDTA) as an anticoagulant. Quantitative indicators of blood cell elements were determined using an automatic hematology analyzer Sysmex-XT, 1800i (Japan). The following parameters were measured: total red blood cell count (RBC), hemoglobin level (HGB), hematocrit (HCT), white blood cell count (WBC), and platelet count (PLT). Biochemical parameters were determined using a semi-automatic biochemical analyzer Urit 800 Vet (China). The parameters studied were: enzyme activity — alanine aminotransferase (ALT), aspartate aminotransferase (AST), total protein, total bilirubin, urea, and cholesterol.

### Histological examination

Preparation of biomaterial. After 14 days of observation, the animals were euthanized by decapitation under light ether anesthesia. A complete autopsy was then performed with removal of internal organs (liver, kidneys, pancreas, heart, spleen) for macroscopic evaluation and further histological studies. The animal tissues were fixed in neutral buffer 10% formalin (Biovitrum, Italy) at +4 °C for 24 h. Tissue sections were prepared according to a standard protocol developed at the University of Washington Veterinary Diagnostic Laboratory (Snyder et al., 2016) to ensure uniformity of sections for all animals.

Liver: a transverse section was made in the left and right medial lobes.

Heart: a longitudinal section was made through both ventricles, from the base to the apex, including the interventricular septum, papillary muscles, and outflow vessels.

Spleen: A transverse section was made in the widest part of the organ.

Each sample was labeled with an individual animal number indicating the corresponding experimental group.

After fixation, the samples were washed with distilled water, sequentially dehydrated in ethanol with increasing concentration, then immersed in xylene and poured into paraffin blocks. Tissue sections 3–4 μm thick were obtained using a microtome and placed on slides. The slides were then deparaffinized and stained with hematoxylin and eosin. Mounting medium was applied to each section, covered with a cover slip, and allowed to dry.

The stained sections were examined by light microscopy. The preparations were examined on a Zeiss AxioLab 4.0 microscope at magnifications of ×40, ×100, ×200, and ×400. ImageJ software (version 1.54p) for Windows was used to photograph the images.

### Morphometric study

The morphometric study was performed by two independent researchers with experience in experimental pathomorphology in animal models, without knowledge of the animal’s group affiliation or the intervention performed.

Lesions found in each organ of the mice were identified and described in accordance with the International Harmonization of Nomenclature and Diagnostic Criteria (INHAND) (Keenan et al., 2015).

A semi-quantitative scale was used to assess the severity of structural and inflammatory changes in tissues.

In the kidneys, liver, heart, spleen, and pancreas, cellular infiltrate, necrosis, and venous congestion were assessed as follows:

no changes - 0 (no discernible changes, 0%); initial changes - 1 (initiation of changes, up to 10%); minimal changes - 2 (focal changes, 11%–25%); moderate changes - 3 (widespread changes, 26%–75%); pronounced changes - 4 (diffuse changes, 76%–90%); severe changes - 5 (widespread changes, 91%–100%) (Lee et al., 2008).

Kidney. In addition, the following morphological features were subjected to binary (dichotomous) assessment: tubular epithelial vacuolization, tubular epithelial cell hyperplasia, protein cylinder formation in the tubules, focal tubular lumen enlargement, glomerulopathy, and glomerular hypertrophy. The presence was indicated by a score of “1”, the absence by “0”.

Liver. Morphological changes were assessed using an adapted histopathological analysis scale described in previously published works (Veteläinen et al., 2006; Niazi et al., 2021).

The following morphological features were additionally subjected to binary (dichotomous) assessment: periportal fibrosis, hyperplasia/cysts of the bile ducts, karyocytomegaly, and/or multinucleated hepatocytes. The presence was indicated by a score of “1”, and the absence by “0”.

Heart. The following morphological features were additionally subjected to binary (dichotomous) assessment: cardiomyocyte hypertrophy, focal hypereosinophilia, and increased cardiomyocyte nucleus size/appearance of multinucleated cardiomyocytes. Presence was indicated by a score of “1”, absence by “0”.

Spleen. The following morphological features were additionally subjected to binary (dichotomous) assessment: lymphoid hyperplasia (increase in the number/density of lymphoid follicles and/or germinal centers), decrease in the number/density of lymphoid follicles and/or germinal centers, atypical forms or pathological enlargement of megakaryocytes. The presence was indicated by a score of “1”, the absence by “0”.

Pancreas. The following morphological features were additionally subjected to binary (dichotomous) assessment: hyperplasia of the islets of Langerhans, atrophy of the islets of Langerhans, interlobular stroma edema, cell vacuolization, karyopyknosis and karyomegaly, and apoptosis. Presence was indicated by a score of “1,” absence by “0.”

### Ethical approval

The study was conducted in accordance with the ethical principles for the treatment of laboratory animals set out in the Declaration of Helsinki and the OECD guidelines. The research protocol was approved by the Bioethics Committee of the Karaganda Medical University (Protocol No. 10 of May 30, 2025, registration number 12).

### Statistical analysis

Statistical analysis was performed using Statistica 10.0 (StatSoft Inc.) and IBM SPSS Statistics 25.0 (IBM Corp., Armonk, New York, United States) software. Quantitative variables were initially analyzed using the Shapiro-Wilk test to determine the normality of distribution, using the Levene test to check the homogeneity of variances. The mean, standard deviation (SD), and 95% confidence interval (95%CI) limits were calculated for normally distributed data. Comparison of categorical variables between groups was performed using χ^2^. Fisher’s exact test was used when there were restrictions on sample size. The Mann-Whitney U test was used to compare independent quantitative variables in cases where there were no signs of normal distribution of data. When comparing mean values, the independent Student’s t-test was calculated to compare two independent groups in normally distributed sets of quantitative data. Differences were considered significant if p < 0.05.

## Results

### Study of the chemical composition of thick extract *I. scariosa*

**FIGURE 1:**
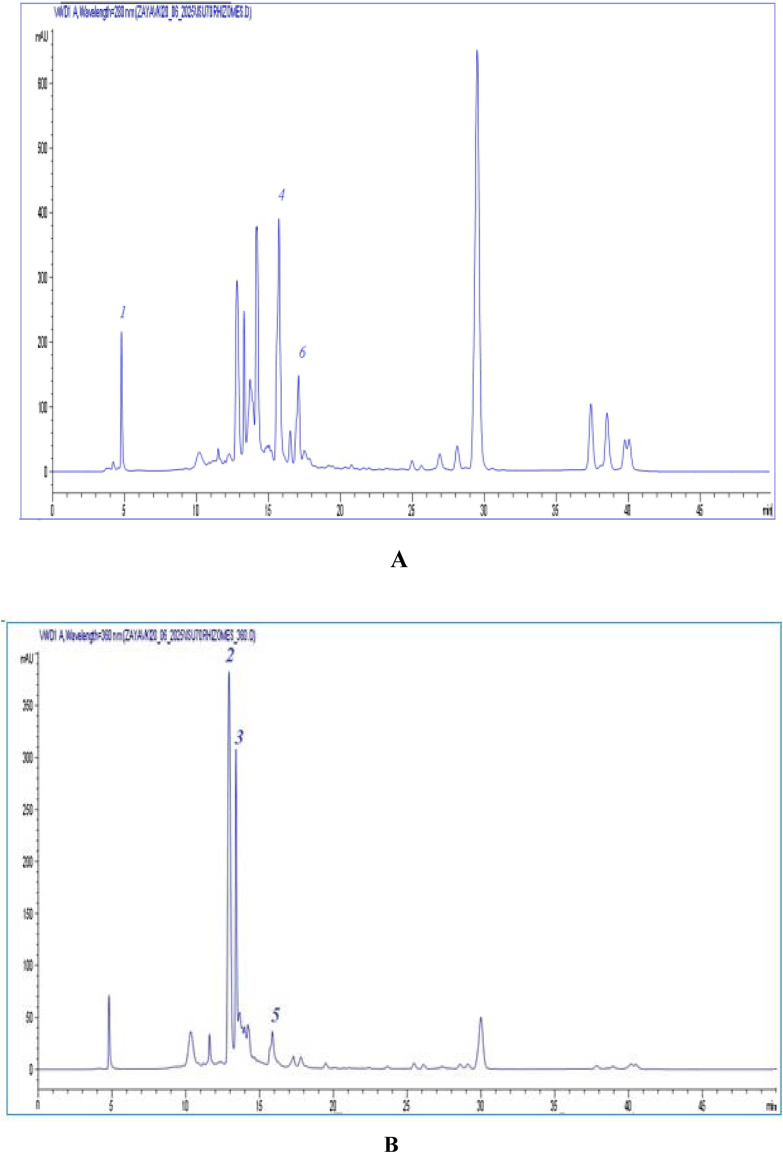
Chromatograms of dense extract *I. scariosa.* **(A)** - UV chromatogram at a wavelength of 280 nm ISU70 rhizomes: gallic acid (1), p-coumaric acid (4), rosmarinic acid (6). **(B)** – UV chromatogram at a wavelength of 360 nm ISU70 rhizomes: catechin (2), epicatechin (3), apigenin-7-O-glucuronide (5).

The chemical composition of phenolic compounds in *I. scariosa* extracts was analyzed at the research center of the Karaganda Medical University (Karaganda, Kazakhstan). Analysis of the chromatographic profiles revealed six dominant peaks with characteristic retention times (tR 5.19–17.01 min) and negative ion mass spectra [M−H]⁻. Comparison of mass spectra, retention times, and UV spectra with literature data and standard substances allowed the identification of the following compounds: gallic acid **(1)**, catechin **(2)**, epicatechin **(3)**, p-coumaric acid **(4)**, apigenin-7-O-glucuronide **(5)**, and rosmarinic acid **(6)** (Table 3). As can be seen from Table 3, the dominant components of *I. scariosa* extract are: p-coumaric acid – 19.23 ± 2.36 mg/g, catechin – 17.07 ± 0.13 mg/g, epicatechin – 13.56 ± 0.21 mg/g. Thus, the predominant compounds are flavan-3-ols (catechin and epicatechin) (Mocan et al., 2020) and p-coumaric acid, which is consistent with the literature data for representatives of the genus Iris, characterized by a high content of phenolic acids and flavonoids (Machalska et al., 2008; Mykhailenko et al., 2020). HPLC-UV chromatograms at 280 nm and 360 nm are shown in Figure 1. Compound **1** was identified as gallic acid with a molecular ion [M–H]^-^ at m/z 169 and a UV absorption maximum at 280 nm for phenolic acids. The gallic acid content was 11.01 ± 0.17 mg/g of extract. Similar spectral characteristics of gallic acid have been previously reported for extracts of I. variegata and I. hungarica (Mykhailenko et al., 2020; Fernandes et al., 2016). Compounds **2** and **3**, corresponding to catechin and epicatechin, showed absorption maxima at 280 nm (with a slight shoulder at ∼ 320 nm), which is characteristic of flavan-3-ols. For both compounds, an ion [M–H]^-^ was recorded at m/z 289. According to the literature, flavan-3-ols exhibit intense UV absorption in the range of 275–285 nm, due to the system of conjugated double bonds in the benzopyran ring, while the shoulder at 320 nm reflects the presence of hydroxyl groups in position 3 (Zakia et al., 2025). Catechin and epicatechin were previously isolated from *I. germanica* L., *I. schachtii* Markgr., and *I. pseudacorus* L., where they acted as biomarkers of proanthocyanidins, key antioxidant components of the genus Iris (Khatib et al., 2022; Mocan et al., 2020). Compound **4**, with tR = 15.79 min and ion [M–H]- = 163, was identified as p-coumaric acid. Its absorption maximum was observed at 280 nm. Coumaric acid is known for its pronounced antioxidant, anti-inflammatory, and antimicrobial properties (Mykhailenko et al., 2020; Chen et al., 2024). Compound **5** with an ion [M–H]^-^ = 445 and a retention time of 15.93 min was identified as apigenin-7-O-glucuronide. Its UV spectrum is characterized by two intense absorption bands: at 270–275 nm (B band, benzene ring B transition) and 335–360 nm (A band, conjugated C–O–B ring system), which is typical for flavones. The wavelength of maximum absorption at 360 nm confirms that the compound belongs to the flavone class (Peng et al., 2016). The content of apigenin-7-O-glucuronide was 2.36 ± 0.13 mg/g of extract. Compound **6** (tR = 17.01 min, [M–H]^-^ = 359) was identified as rosmarinic acid, known as dihydroxycinnamic acid with a maximum absorption at 280–330 nm. The content of rosmarinic acid was 6.43 ± 0.60 mg/g of extract.

**TABLE 3:** Identified phytochemical constituents extract of *I. scariosa*, rhizomes.

No	RT (min)	[М-Н]^-^(m/z)	Identified compounds	Content (mg/g of extract)	UV λmax (nm)
1	5.19	169	Gallic acid	11.01 ± 0.17	280
2	13.32	289	Catechin	17.07 ± 0.13	280
3	13.94	289	Epicatechin	13.56 ± 0.21	280
4	15.79	163	p-Coumaric acid	19.23 ± 2.36	280
5	15.93	445	Apigenin-7-O-glucuronide	2.36 ± 0.13	360
6	17.01	359	Rosmarinic acid	6.43 ± 0.6	280

### Acute toxicity study

During 14 days of observation after a single intragastric administration of *I. scariosa* L. extract, no fatalities were observed in animals in any of the experimental groups (Table 4). The general condition of the mice was assessed as satisfactory. All individuals maintained a normal appearance: their fur was clean, smooth, with no signs of hair loss or contamination; their skin and mucous membranes had a physiological color. Motor activity was within the age norm, with no signs of hypo- or hyperactivity.

**TABLE 4:** Dose-dependent mortality in mice after a single administration of *I. scariosa* L. Extract.

Group name	Gender (M/F)	Dose (mg/kg)	Number of animals (n)	Number of deaths	Percentage of dead animals %
A	M	500	4	0	0
	F		4	0	0
B	M	1,000	4	0	0
	F		4	0	0
C	M	2000	4	0	0
	F		4	0	0
Control	M	Distilled water	4	0	0
	F		4	0	0

In all groups, the mice responded to auditory and tactile stimuli and showed interest in food and water. No abnormalities in urination or defecation were observed. During daily visual inspection, no signs of tremors, convulsions, muscle rigidity, coordination disorders, pathological postures, or self-injury were detected. No symptoms characteristic of Straube’s syndrome were observed. The animals’ breathing was even, with no signs of depression or shortness of breath. There were no symptoms of sedation, diarrhea, or loss of consciousness. The body weight of the animals was recorded at baseline and on days 1, 5, 10, and 14 of the experiment. As shown in Table 5, all groups showed positive body weight gain without sharp fluctuations or growth delays. In mice receiving Iris scariosa L. extract at a dose of 500 mg/kg (n = 8), body weight was slightly lower than in the control group (n = 8) at all time points, but the differences were not statistically significant (p > 0.05). On day 14, the average body weight was 33.44 ± 2.47 g compared to 35.70 ± 2.12 g in the control group. Despite the observed trend toward slower body weight gain, the differences did not reach statistical significance throughout the observation period (p > 0.05 at all time points). This indicates that the administration of the extract at a dose of 500 mg/kg does not cause a significant decrease in body weight.

**TABLE 5:** Change in body weight of animals after a single administration of *I. scariosa* L. Extract.

Average weight of animals (g)					
Doses (mg/kg)	Initial mass	1 day	5 days	10 days	14 days
500	31.06 ± 2.61	31.52 ± 2.67	32.12 ± 2.54	32.74 ± 2.59	33.44 ± 2.47
1,000	32.30 ± 1.72	32.76 ± 1.65	33.58 ± 2.07	34.26 ± 1.43	34.64 ± 1.59
2000	33.96 ± 2.16	34.32 ± 2.20	35.10 ± 2.12	35.46 ± 2.25	35.94 ± 2.41
Control	33.62 ± 2.59	34.06 ± 2.12	34.46 ± 1.94	35.08 ± 2.08	35.70 ± 2.12
p-value	0.4532	0.4343	0.4272	0.4782	0.4763

In animals receiving *I. scariosa* L. extract at a dose of 1,000 mg/kg (n = 8), body weight remained comparable to that of the control group throughout the observation period. On day 14, the average body weight was 34.64 ± 1.59 g, which was slightly lower than the control value (35.70 ± 2.12 g), while the observed differences did not reach statistical significance at all stages of the study (p = 0.31–0.51). Student’s t-test values ranged from −0.69 to −1.08, indicating no significant differences between the experimental and control groups. These data indicate that this dose of extract had no negative effect on growth and metabolism.

Against the background of the administration of *I. scariosa* L. extract at a dose of 2000 mg/kg (n = 8), the body weight of mice at all stages was comparable to or slightly higher than that of the control group. By day 14, the average weight was 35.94 ± 2.41 g, which is slightly higher than the control value, but no statistically significant difference was recorded (p = 0.871). Thus, the maximum tested dose did not cause disturbances in body weight dynamics and, on the contrary, may have a slight positive physiological effect.

**FIGURE 2:**
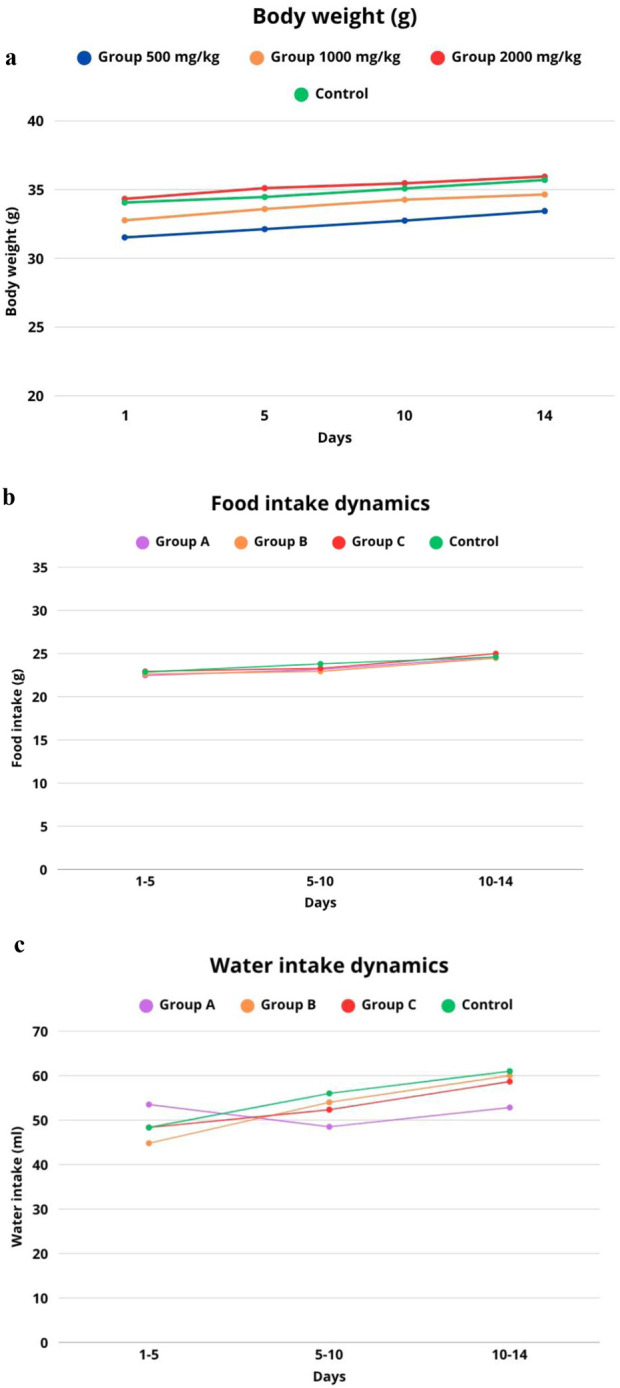
Dynamics of parameters in laboratory mice after a single administration of *I.scariosa* L. extract **(a)** change in body weight, **(b)** food consumption, **(c)** water consumption during a 14-day observation period.

At the same time, food and water consumption were monitored as important parameters of metabolic and behavioral status. The summarized data on body weight dynamics, food and water consumption are presented in Figure 2. As can be seen from the graph, all experimental groups showed positive body weight gain dynamics (Figure 2a).

The feeding behaviour of animals after a single administration of *I. scariosa* L. extract in the control group (n = 8) remained stable throughout the 14-day observation period. During days 1–5, the average consumption was 22.86 ± 0.82 g, and on days 5–10, there was a slight increase in consumption, averaging 23.81 ± 1.19 g, which may reflect the natural growth and adaptation of the animals. On days 10–14, the consumption level stabilised at 24.61 ± 0.49 g, indicating that the animals maintained a normal physiological state (Figure 2b).

In the group of animals receiving *I. scariosa* L. at a dose of 500 mg/kg (n = 8), no significant disturbances in eating behaviour were observed. On days 1–5, food consumption was 22.46 ± 0.59 g, close to the control (p = 0.317), indicating the absence of early anorexia. On days 5–10, there was a moderate increase in consumption to 23.16 ± 0.53 g, but the difference with the control was statistically insignificant (p = 0.216). On days 10–14, consumption reached 24.61 ± 0.90 g, completely coinciding with the values of the control group (p = 1.000).

Animals receiving *I. scariosa* L. extract at a dose of 1,000 mg/kg (n = 8) maintained stable food consumption throughout the observation period. On days 1–5, the average value was 22.63 ± 0.66 g, with no significant difference from the control (p = 0.577). On days 5–10, consumption decreased to 22.94 ± 0.56 g, which was slightly lower than the control values but did not reach statistical significance (p = 0.115). On days 10–14, the average consumption was 24.47 ± 0.62 g, comparable to the control level (p = 0.643).

In animals that received the maximum possible dose of Iris scariosa L. extract 2000 mg/kg (n = 8), no signs of appetite suppression or food intake disorders were detected. On days 1–5, the average value was 22.94 ± 0.32 g, comparable to the control group (p = 0.804). On days 5–10, food consumption was 23.29 ± 0.60 g (p = 0.322), with no significant deviations. Between days 10 and 14, there was even a slight increase.

Water consumption in laboratory animals after a single intragastric administration of *I. scariosa* L. extract in the control group (n = 8) averaged 48.33 ± 6.62 mL on days 1–5. The animals showed a stable level of water consumption with a tendency towards a moderate increase by day 14, which corresponds to normal physiological recovery after manipulation (Figure 2c). In the group of animals receiving *I. scariosa* L. extract at a dose of 500 mg/kg (n = 8), the following changes in water consumption were observed during the 14-day observation period. During the first 5 days, the average water consumption was 53.50 ± 5.58 mL, which is slightly higher than the corresponding indicator in the control group. However, statistical analysis did not reveal any significant differences (p = 0.175), which may indicate the preservation of the physiological norm of water metabolism in the early stages after the administration of the extract. On days 5–10, a significant decrease in water consumption to 48.50 ± 4.37 mL was noted compared to the control (p = 0.014). This decrease may be associated with the body’s adaptive response to the presence of bioactive compounds of plant origin that affect metabolism or behavioural mechanisms of water balance regulation. Between days 10 and 14, water consumption was 52.83 ± 7.17 mL, which was also significantly lower than the control values (p = 0.022). At a dose of 1,000 mg/kg, water consumption did not differ significantly from the control values, which may indicate the absence of a pronounced toxic effect on water metabolism parameters.

In the group of animals receiving Iris scariosa L. extract at a dose of 2000 mg/kg (n = 8), the dynamics of water consumption over 14 days was characterised by the absence of pronounced deviations from the control values. During the first 5 days, the average water consumption was 48.33 ± 4.08 mL, which is practically identical to the control group (p = 1.0), indicating the absence of early toxicological effects affecting thirst or water-salt balance. On days 5–10, the average water consumption was 52.33 ± 3.20 mL, which is slightly lower than the control value, but the difference was not statistically significant (p = 0.132). Between days 10 and 14, water consumption increased to 58.67 ± 3.72 mL. Thus, the administration of *I. scariosa* L. extract at a dose of 2000 mg/kg did not cause any significant changes in water consumption indicators throughout the observation period.

### Study of haematological and biochemical parameters

Haematological parameters reflect the general physiological state of the body and allow the identification of possible toxic effects on the haematopoietic system. As part of this study, a comparison was made of blood parameters in laboratory mice after a single intragastric administration of *I. scariosa* L. extract at doses of 500, 1,000 and 2000 mg/kg compared to the control group. In animals receiving the extract at a dose of 500 mg/kg, the levels of haemoglobin, erythrocytes, leukocytes, haematocrit, erythrocyte volume, haemoglobin concentration and thrombocytes were within the physiological norm (Table 6) and did not differ from the control group (p > 0.05). Although there was a tendency toward a slight increase in haemoglobin (15.1 ± 0.4 g/dL vs. 14.7 ± 0.4 g/dL) and red blood cell count (8.9 ± 0.3 vs. 7.7 ± 0.5 × 10^12^/L), the differences were not statistically significant (p = 0.09 and p = 0.08, respectively), which may reflect individual variations in physiological values.

**TABLE 6:** Haematological parameters of laboratory mice.

Parameters	Doses (mg/kg)			
	500 mg/kg	1,000 mg/kg	2000 mg/kg	Control mg/kg
HGB (g/dL)	15.1 ± 0.4	15.4 ± 0.3	14.8 ± 0.5	14.7 ± 0.4
WBC (109 L)	7.8 ± 0.6	8.1 ± 0.5	10.3 ± 0.7	9.6 ± 0.6
RBC (1012L)	8.9 ± 0.3	9.1 ± 0.4	8.3 ± 0.4	7.7 ± 0.5
HCT (%)	45.3 ± 1.5	46.2 ± 1.2	45.8 ± 1.4	42.5 ± 1.3
MCV (f)	50.9 ± 1.2	50.7 ± 1.1	51.2 ± 1.3	50.5 ± 1.2
MCH (pg)	16.3 ± 0.4	16.5 ± 0.3	16.8 ± 0.5	16.2 ± 0.4
MCHC (g/dL)	32.1 ± 0.6	32.6 ± 0.5	33.1 ± 0.4	32.0 ± 0.5
PLT (109 L)	880 ± 75	910 ± 68	990 ± 80	925 ± 70
PCT (%)	0.30 ± 0.03	0.31 ± 0.02	0.34 ± 0.03	0.30 ± 0.02

In mice receiving the extract at a dose of 1,000 mg/kg, HGB, RBC, WBC, HCT, MCV, and PLT values remained within normal limits and did not differ from the control group (p > 0.05; see Table 6). The mean concentration of haemoglobin in erythrocytes (MCHC) was slightly higher (32.6 ± 0.5 vs. 32.0 ± 0.5 g/dL), but the difference was not statistically significant (p = 0.067). These results indicate that the extract had no significant effect on the hematopoietic system at this dosage. In females receiving 2000 mg/kg of the extract, no statistically significant changes in most hematological parameters (HGB, RBC, WBC, HCT, MCV, MCH, PLT) compared to the control group (p > 0.05), indicating no pronounced toxic effect on the hematopoietic system. However, a moderate increase in MCHC (33.1 ± 0.4 vs. 32.0 ± 0.5 g/dL; p = 0.0054) and PCT (0.34% ± 0.03% vs. 0.30% ± 0.02%; p = 0.0423). These changes remained within the physiological norm and probably reflect compensatory adaptive responses.

Blood biochemical parameters are presented in Table 7. The results obtained indicate that most of the parameters studied in animals receiving *I. scariosa* L. extract did not have statistically significant differences compared to the control group (p > 0.05), indicating the absence of a pronounced hepatotoxic effect of the test substance when administered once at doses up to 2000 mg/kg.

**TABLE 7:** Biochemical parameters of blood in laboratory mice.

Parameters	Doses (mg/kg)			
	500 mg/kg	1,000 mg/kg	2000 mg/kg	Control mg/kg
Total protein (g/dL)	65.8 ± 2.7	66.6 ± 2.5	67.4 ± 2.8	66.2 ± 2.3
Urea, mmol/L	7.0 ± 0.6	7.4 + 0.5	8.6 ± 0.6	6.8 + 0.7
Glucose, mmol/L	6.36 ± 0.25	6.4 ± 0.6	6.2 ± 0.8	6.5 ± 0.22
Total bilirubin (mg/dL)	2.5 ± 0.3	2.7 ± 0.4	2.8 ± 0.5	2.4 ± 0.2
ALT (SGPT) (U/L)	37.6 ± 4.7	41.3 ± 5.4	45.1 ± 6.0	37.4 ± 4.5
AST (SGOT) (U/L)	122.8 ± 11.3	126.7 ± 12.5	134.2 ± 13.0	121.0 ± 10.2
HS, mmol/L	1.4 ± 0.2	1.6 ± 0.2	1.6 ± 0.3	1.4 ± 0.1

Total protein values varied within the physiological norm (65.8 ± 2.7–67.4 ± 2.8 g/L) and did not differ statistically significantly from the control group (66.2 ± 2.3 g/L), indicating no pronounced protein imbalance or impaired synthetic function of the liver. Urea levels showed a dose-dependent increase: from 7.0 ± 0.6 mmol/L at 500 mg/kg to 8.6 ± 0.6 mmol/L at 2000 mg/kg, which may indicate an increased load on kidney function. Glucose levels remained stable (6.2–6.5 mmol/L), close to the control values (6.5 ± 0.22 mmol/L), indicating no hypo- or hyperglycaemic effect of the extract.

Total bilirubin levels showed a slight increase in the experimental groups (up to 2.8 ± 0.5 μmol/L at 2000 mg/kg) compared to the control group (2.4 ± 0.2 μmol/L), however, these values did not exceed physiological limits and were not statistically significant.

At the maximum dose (2000 mg/kg), there was a tendency towards a moderate increase in liver enzyme activity. Thus, ALT (SGPT) and AST (SGOT) levels reached 45.1 ± 6.0 and 134.2 ± 13.0 U/L, respectively, exceeding the values of the control group (37.4 ± 4.5 and 121.0 ± 10.2 U/L). However, these changes were not statistically significant (p = 0.08 and p = 0.07), which may indicate a compensatory reaction of the liver without signs of pronounced cytolysis.

Thus, the analysis of biochemical parameters indicates the absence of significant toxic effects on the liver and kidneys, even at the maximum dose, with possible slight adaptive changes that do not exceed physiological limits.

### Analysis of histopathological changes in internal organs in the study groups

#### Kidneys

A comparative histomorphometric characterisation of changes in kidney tissue in the study groups stained with haematoxylin and eosin is presented in Table 8.

**TABLE 8:** Histopathological assessment of renal alterations.

Histological criteria	Study groups n = 32								p-value
	Control group (n = 8)		*I. scariosa* L. 500 mg/kg (n = 8)		*I. scariosa* L. 1,000 mg/kg (n = 8)		*I. scariosa* L. 2000 mg/kg (n = 8)		
	M±SD		M±SD		M±SD		M±SD		
Cellular infiltrate	0.0 ± 0.0		0.6 ± 0.7		1.0 ± 0.5		3.1 ± 0.8		p_1_ = 0.105 p_2_ = **0.002** p_3_ = **0.0001**
Necrosis	0.0 ± 0.0		0.5 ± 0.5		0.4 ± 0.5		1.0 ± 0.5		p_1_ = 0.105 p_2_ = 0.234 p_3_ = **0.002**
Venous congestion	0.0 ± 0.0		1.0 ± 0.8		1.0 ± 0.5		2.3 ± 0.5		p_1_ = **0.010** p_2_ = **0.002** p_3_ = **0.0001**
^a^ n (%)	0	1	0	1	0	1	0	1	
Tubular epithelial vacuolization	8 (100%)	0 (0%)	7 (87.5%)	1 (12.5%)	6 (75%)	2 (25%)	3 (37.5%)	5 (62.5%)	p_1_ = 0.302 p_2_ = 0.450 p_3_ = **0.008**
Tubular epithelial cell hyperplasia	8 (100%)	0 (0%)	8 (100%)	0 (0%)	6 (75%)	2 (25%)	1 (12.5%)	7 (87.5%)	p_1_ = 1.00 p_2_ = 0.450 p_3_ = **0.0001**
Formation of protein casts in tubules	8 (100%)	0 (0%)	8 (100%)	0 (0%)	5 (62.5%)	3 (37.5%)	1 (12.5%)	7 (87.5%)	p_1_ = 1.00 p_2_ = 0.201 p_3_ = **0.0001**
Focal tubular lumen dilatation	8 (100%)	0 (0%)	6 (75%)	2 (25%)	4 (50%)	4 (50%)	0 (0%)	8 (100%)	p_1_ = 0.450 p_2_ = 0.084 p_3_ = **0.000**
Glomerulopathy (focal hyalinosis, mesangial proliferation)	8 (100%)	0 (0%)	8 (100%)	0 (0%)	7 (87.5%)	1 (12.5%)	4 (50%)	4 (50%)	p_1_ = 1.00 p_2_ = 0.302 p_3_ = 0.084
Glomerular hypertrophy	8 (100%)	0 (0%)	8 (100%)	0 (0%)	7 (87.5%)	1 (12.5%)	6 (75%)	2 (25%)	p_1_ = 1.00 p_2_ = 0.302 p_3_ = 0.450

^a^ “0” - absence, “1” - presence.

p_1_ < 0.05 – statistically significant differences between the Iris scariosa L. group (500 mg/kg) and the control group.

p_2_ < 0.05 – statistically significant differences between the Iris scariosa L. group (1,000 mg/kg) and the control group.

p_3_ < 0.05 – statistically significant differences between the Iris scariosa L. group (2000 mg/kg) and the control group.

Statistically significant values (p < 0.05) are highlighted in bold.

**FIGURE 3:**
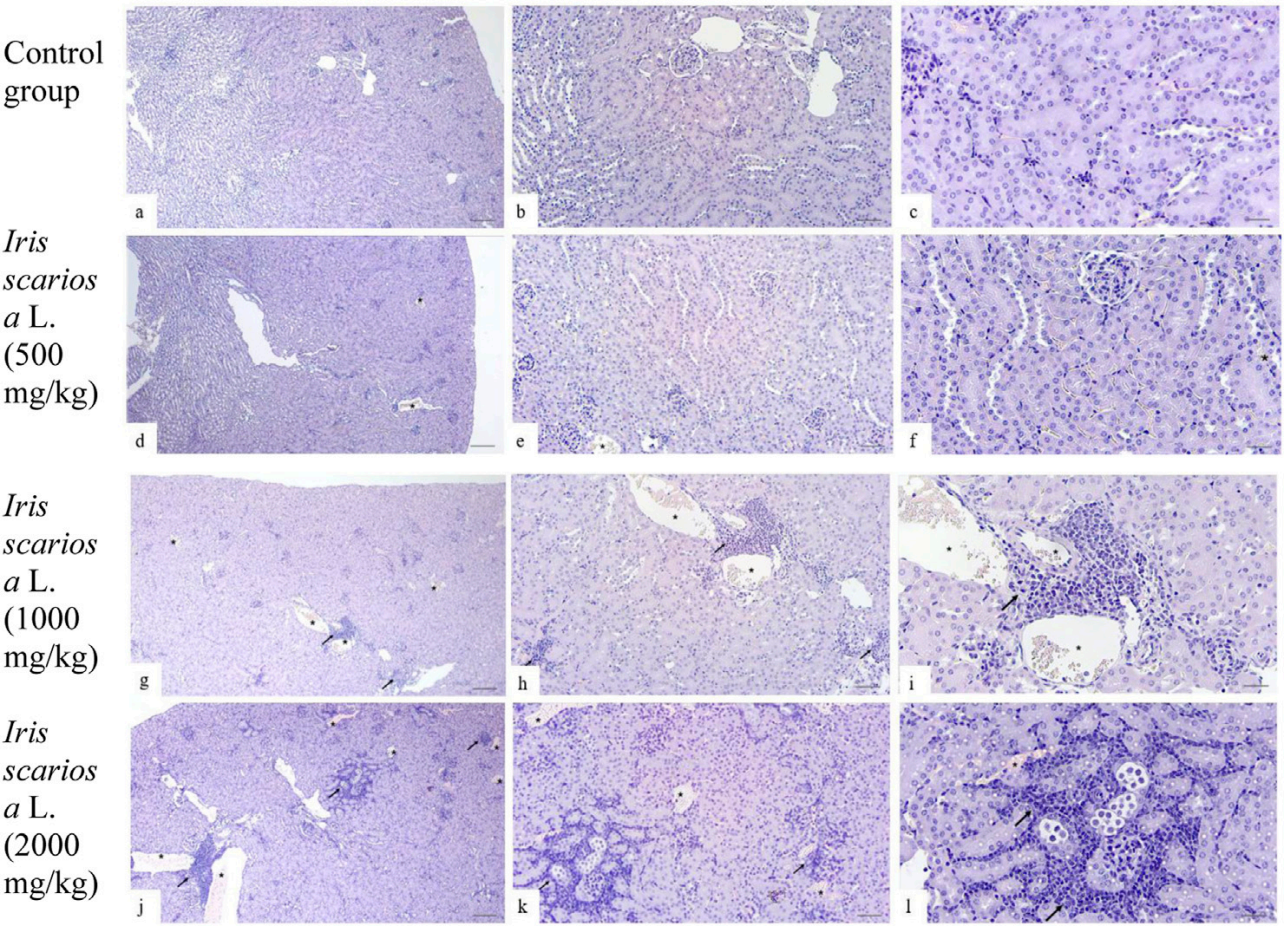
Representative microscopic sections of kidneys. H&E Legend: * - venous congestion, black arrow - cellular infiltration. **(a–c)** - Control group. The histological structure of the kidney corresponds to the histological norm. The glomeruli are compact, the capillary loops are evenly expressed, the lumens are preserved, with no signs of hyperemia, mesangial proliferation, or sclerosis. The proximal convoluted tubules are lined with tall single-layered pyramidal epithelium. The distal convoluted tubules have a relatively wide lumen, lined with cubic epithelium with light, moderately acidophilic cytoplasm and rounded nuclei. **(a)** - x40, **(b)** - x100, **(c)** - x200. **(d–f)** - *I. scariosa* L., 500 mg/kg. **(d)** - acute venous hyperemia. x40. **(e)** - partial reduction of capillary loops with signs of compaction and moderate interstitial edema with expansion of the intercellular space. x100. **(f)** - proximal tubule epithelium swollen in places, with compacted cytoplasm, histopathological signs of vacuolar dystrophy. In the distal convoluted tubules, the cubic epithelium is preserved, but there are areas with enlarged lumens. x200. **(g–i)** *I. scariosa* L., 1,000 mg/kg **(g)** - mild tubulointerstitial nephritis. x40. **(h)** - foci of lymphohistiocytic infiltration, located mainly perivascularly and periglomerularly, as well as in the interstitium between the tubules. x100. **(i)** - Focal accumulations of inflammatory cells, mainly lymphocytes and macrophages. The vascular wall and tubule epithelium are preserved. x200. **(j–l)** – *I. scariosa* L., 2000 mg/kg. **(j)** - pronounced tubulointerstitial nephritis. x40. **(k)** - multiple areas of inflammatory infiltration, including clusters of lymphoid cells in the interstitium between the tubules. x100. **(l)** - pronounced pericanalicular lymphocytic and macrophage infiltration. The structure of the tubules is partially disrupted, there is a change in the tinctorial properties of the cells, part of the epithelium is flattened and desquamated. x200.

In the control group, the structure of the kidneys was normal: the glomeruli had a typical structure, the tubules were lined with homogeneous epithelium without signs of destruction, vacuolization, or hyperplasia; the lumens were not dilated, and there was no infiltration or protein casts (Figures 3a–c).

In the group of animals receiving *I. scariosa* at a dose of 500 mg/kg, the histoarchitecture was preserved. Half of the animals showed initial signs of tubular and vascular damage: diffuse lymphohistiocytic infiltration (0.6 ± 0.7) and mild venous stasis (1.0 ± 0.8). In a few animals, mild dystrophic changes in the tubular epithelium were noted: vacuolization (12.5%) and focal lumen enlargement (25%). (Figures 3d–f).

At a dose of 1000 mg/kg, signs of mild tubulointerstitial damage were detected: foci of perivascular and periglomerular infiltration (1.0 ± 0.5 points), isolated cellular necrosis (0.4 ± 0.5 points), and mild venous stasis (1.0 ± 0.5 points). In some animals, vacuolization and hyperplasia of the tubular epithelium, protein casts (37.5%), and focal dilation of the tubular lumen (50%) were observed (Figures 3g–i).

At a dose of 2000 mg/kg, most animals showed signs of moderate tubulointerstitial damage. In all cases, inflammatory infiltration was observed in the stroma, represented by clusters of lymphocytes and plasma cells in the interstitium between the tubules (3.1 ± 0.8). In 3 (37.5%) cases, pronounced changes were observed, in 3 (37.5%) - moderate changes, and in 2 (25%) - minimal changes. No severe or moderate necrotic changes were observed, but 7 (87.5%) animals had initial and focal necrosis (1.0 ± 0.5 points). In all cases, signs of venous stasis were observed: in 6 (75%) cases of minimal degree, in 2 (25%) cases of moderate degree. The average score for venous stasis in the group of animals was 2.3 ± 0.5 points. In 5 (62.5%) mice, vacuolization of the tubular epithelium was observed, in 7 (87.5%) hyperplasia of tubular epithelial cells and the formation of protein cylinders in their lumen were noted. All animals showed focal enlargement of the tubular lumen. Glomerulopathy was detected in 4 (50%) cases, and glomerular hypertrophy in 2 (25%) mice (Figures 3j–l).

#### Liver

A comparative histopathological assessment of liver damage in mice in the study groups is presented in Table 9.

**TABLE 9:** Histopathological assessment of liver changes.

Histological criteria	Study groups n = 32								p-value
	Control group (n = 8)		*I. scariosa* L. 500 mg/kg (n = 8)		*I. scariosa* L. 1,000 mg/kg (n = 8)		*I. scariosa* L. 2000 mg/kg (n = 8)		
	M±SD		M±SD		M±SD		M±SD		
Cellular infiltrate	0.0 ± 0.0		0.3 ± 0.5		0.6 ± 0.7		2.0 ± 0.5		p_1_ = 0.442 p_2_ = 0.105 p_3_ = **0.0001**
Necrosis	0.0 ± 0.0		0.3 ± 0.5		0.4 ± 0.5		0.4 ± 0.5		p_1_ = 0.442 p_2_ = 0.234 p_3_ = 0.234
Venous congestion	0.0 ± 0.0		0.6 ± 0.5		0.8 ± 0.7		1.9 ± 0.8		p_1_ = **0.038** p_2_ = **0.038** p_3_ = **0.0001**
^a^ n (%)	0	1	0	1	0	1	0	1	
Periportal fibrosis	8 (100%)	0 (0%)	8 (100%)	0 (0%)	8 (100%)	0 (0%)	8 (100%)	0 (0%)	p_1_ = 1.00 p_2_ = 1.00 p_3_ = 1.00
Bile duct hyperplasia/cysts	8 (100%)	0 (0%)	8 (100%)	0 (0%)	8 (100%)	0 (0%)	8 (100%)	0 (0%)	p_1_ = 1.00 p_2_ = 1.00 p_3_ = 1.00
Karyocytomegaly and/or multinucleated hepatocytes	8 (100%)	0 (0%)	8 (100%)	0 (0%)	7 (87.5%)	1 (12.5%)	1 (12.5%)	7 (87.5%)	p_1_ = 1.00 p_2_ = 0.302 p_3_ = **0.0001**
Hepatocyte vacuolization	8 (100%)	0 (0%)	7 (87.5%)	1 (12.5%)	6 (75%)	2 (25%)	4 (50%)	4 (50%)	p_1_ = 0.302 p_2_ = 0.131 p_3_ = **0.021**

^a^ “0” - absence, “1” - presence.

p_1_ < 0.05 – statistically significant differences between the Iris scariosa L. group (500 mg/kg) and the control group.

p_2_ < 0.05 – statistically significant differences between the Iris scariosa L. group (1,000 mg/kg) and the control group.

p_3_ < 0.05 – statistically significant differences between the Iris scariosa L. group (2000 mg/kg) and the control group.

Statistically significant values (p < 0.05) are highlighted in bold.

**FIGURE 4:**
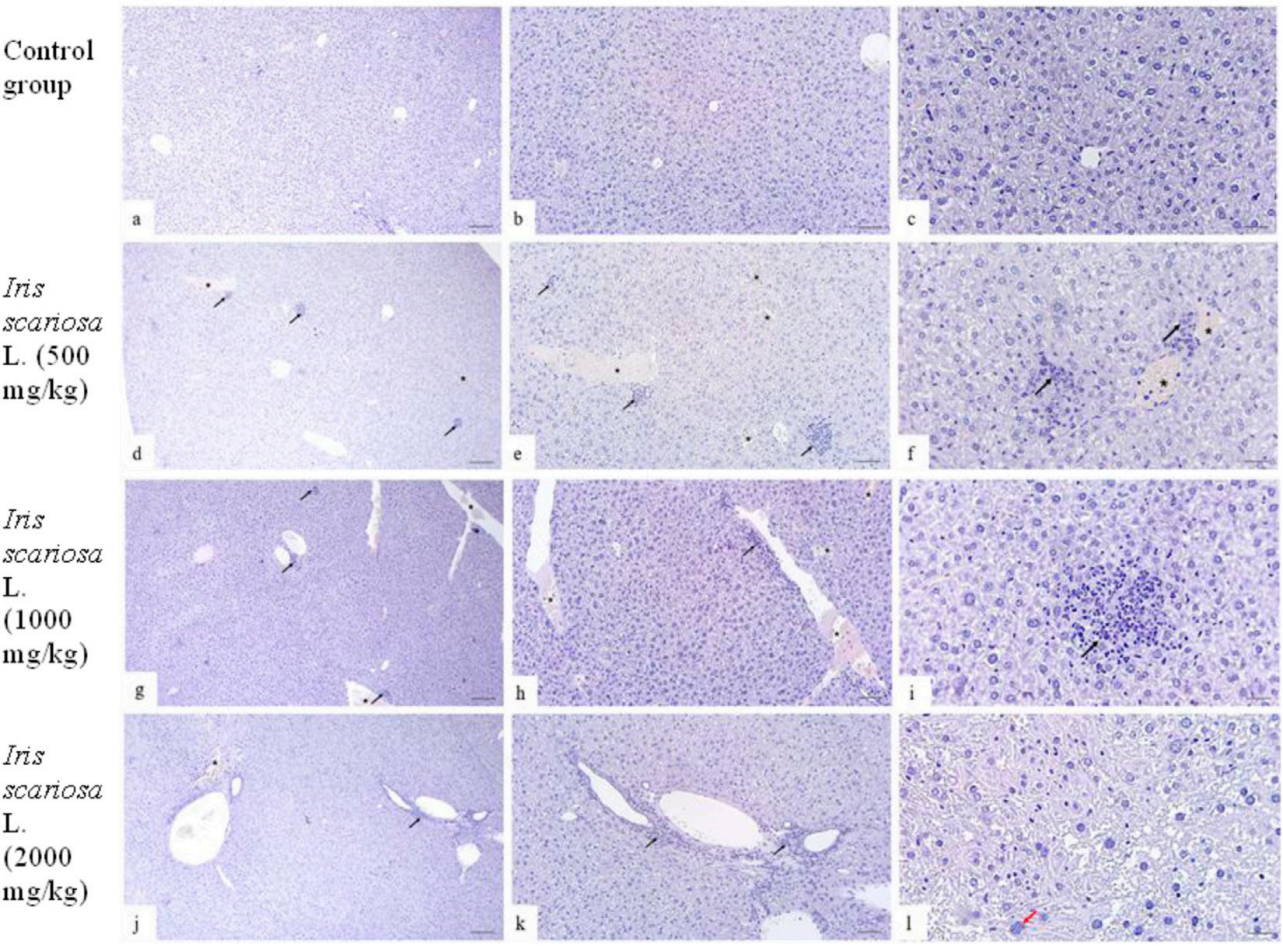
Representative microscopic sections of the liver. H&E Legend: * - venous congestion, black arrow - cellular infiltration, red arrow - multinucleated hepatocyte. **(a–c)** – Control group. The structure of the liver corresponds to the histological norm. Hepatocytes are clearly defined, represented by polygonal cells with centrally located rounded nuclei, well-distinguishable nucleoli, and homogeneous eosinophilic cytoplasm. The tissue is intact without inflammatory infiltration or fibrotic changes. Sinusoids are distinguishable, lumens are not dilated, and there are no signs of stasis. **(a)** - x40, **(b)** - x100, **(c)** - x200. **(d–f)** *I. scariosa* L., 500 mg/kg. Focal venous hyperemia and focal accumulations of lymphocytes and plasma cells. **(d)** - x40, **(e)** - x100, **(f)** - x200. **(g–i)** - *I. scariosa* L., 1,000 mg/kg. **(g)** - mild vascular and structural changes: venous hyperemia with mild cellular infiltration. x40. **(h)** - diffuse perivascular lymphoplasmacytic infiltration and venous hyperemia. x100. **(i)** - Focal clusters of inflammatory cells, mainly lymphocytes and macrophages. x200. **(j–l)** – *I. scariosa* L., 2000 mg/kg. **(j)** - mild venous congestion with lymphohistiocytic infiltration. x40. **(k)** - focal clusters of inflammatory cells are detected in the liver tissue. x100. **(l)** - dystrophic changes in hepatocytes with karyopyknosis and karyorexis of nuclei. x200.

In the control group, the histological structure of the liver in all animals corresponded to the anatomical norm: the lobular structure was preserved, hepatocytes were polygonal in shape with clear boundaries and vesicular nuclei, sinusoids were not dilated, portal tracts were intact, and there was no inflammatory infiltration or fibrosis (Figures 4a-c).

In the group of animals receiving *I. scariosa* at a dose of 500 mg/kg, the histoarchitecture of the liver was generally preserved. Some animals showed mild signs of congestive and inflammatory changes: focal lymphohistiocytic infiltration (0.3 ± 0.5 points), isolated hepatocyte necrosis (0.3 ± 0.5 points), and initial venous congestion (0.6 ± 0.5 points). In one case (12.5%), mild dystrophic changes in hepatocytes were noted (Figures 4d–f).

At a dose of 1000 mg/kg, focal congestive and inflammatory changes were detected: diffuse lymphoplasmacytic infiltration (0.6 ± 0.7 points), isolated cellular necrosis (0.4 ± 0.5 points), and mild venous stasis (0.8 ± 0.7 points). One animal (12.5%) had multinucleated hepatocytes, and two (25%) had mild vacuolization of the hepatocyte cytoplasm (Figures 4g–i).

At a dose of 2000 mg/kg, mild inflammatory and vascular changes were detected in the liver of experimental animals. All animals showed diffuse lymphoplasmacytic infiltration: in 1 (12.5%) - initial, in 6 (75%) - mild, and in 1 (12.5%) case moderate changes. Foci of inflammatory cells (lymphocytes and macrophages) were localized mainly around the portal tracts and small vessels. The average score for inflammatory infiltration in the group of animals was 2.0 ± 0.5 points. In 3 (37.5%) animals, isolated hepatocyte necrosis was detected (0.4 ± 0.5 points). Signs of venous stasis were observed in all animals: in 3 (37.5%) cases, initial and mild changes, in 2 (25%) cases, moderate changes (0.8 ± 0.7 points) . In most animals (87.5%) cases, an increase and hyperchromia of hepatocyte nuclei were noted, and in half of the animals, isolated microvesicular vacuoles in the cytoplasm (Figures 4j–l).

#### Heart

A comparative histopathological assessment of myocardial damage in mice in the study groups is presented in Table 10.

**TABLE 10:** Histopathological assessment of cardiac changes.

Histological criteria	Study groups n = 32								p-value
	Control group (n = 8)		*I. scariosa* L. 500 mg/kg (n = 8)		*I. scariosa* L. 1,000 mg/kg (n = 8)		*I. scariosa* L. 2000 mg/kg (n = 8)		
	M±SD		M±SD		M±SD		M±SD		
Cellular infiltrate	0.0 ± 0.0		0.0 ± 0.0		0.3 ± 0.5		0.4 ± 0.5		p_1_ = 1.00 p_2_ = 0.442 p_3_ = 0.234
Necrosis	0.0 ± 0.0		0.0 ± 0.0		0.0 ± 0.0		0.0 ± 0.0		p_1_ = 1.00 p_2_ = 1.00 p_3_ = 1.00
Venous congestion	0.0 ± 0.0		0.5 ± 0.5		0.9 ± 0.6		1.1 ± 0.8		p_1_ = 0.105 p_2_ = **0.010** p_3_ = **0.010**
^a^ n (%)	0	1	0	1	0	1	0	1	
Cardiomyocyte hypertrophy	8 (100%)	0 (0%)	8 (100%)	0 (0%)	8 (100%)	0 (0%)	8 (100%)	0 (0%)	p_1_ = 1.00 p_2_ = 1.00 p_3_ = 1.00
Focal hypereosinophilia	8 (100%)	0 (0%)	8 (100%)	0 (0%)	8 (100%)	0 (0%)	7 (87.5%)	1 (12.5%)	p_1_ = 1.00 p_2_ = 1.00 p_3_ = 0.302
Enlargement of cardiomyocyte nuclei/presence of multinucleated cardiomyocytes	8 (100%)	0 (0%)	8 (100%)	0 (0%)	7 (87.5%)	1 (12.5%)	3 (37.5%)	5 (62.5%)	p_1_ = 1.00 p_2_ = 0.302 p_3_ = **0.031**

^a^ “0” - absence, “1” - presence.

p_1_ < 0.05 – statistically significant differences between the Iris scariosa L. group (500 mg/kg) and the control group.

p_2_ < 0.05 – statistically significant differences between the Iris scariosa L. group (1,000 mg/kg) and the control group.

p_3_ < 0.05 – statistically significant differences between the Iris scariosa L. group (2000 mg/kg) and the control group.

Statistically significant values (p < 0.05) are highlighted in bold.

**FIGURE 5:**
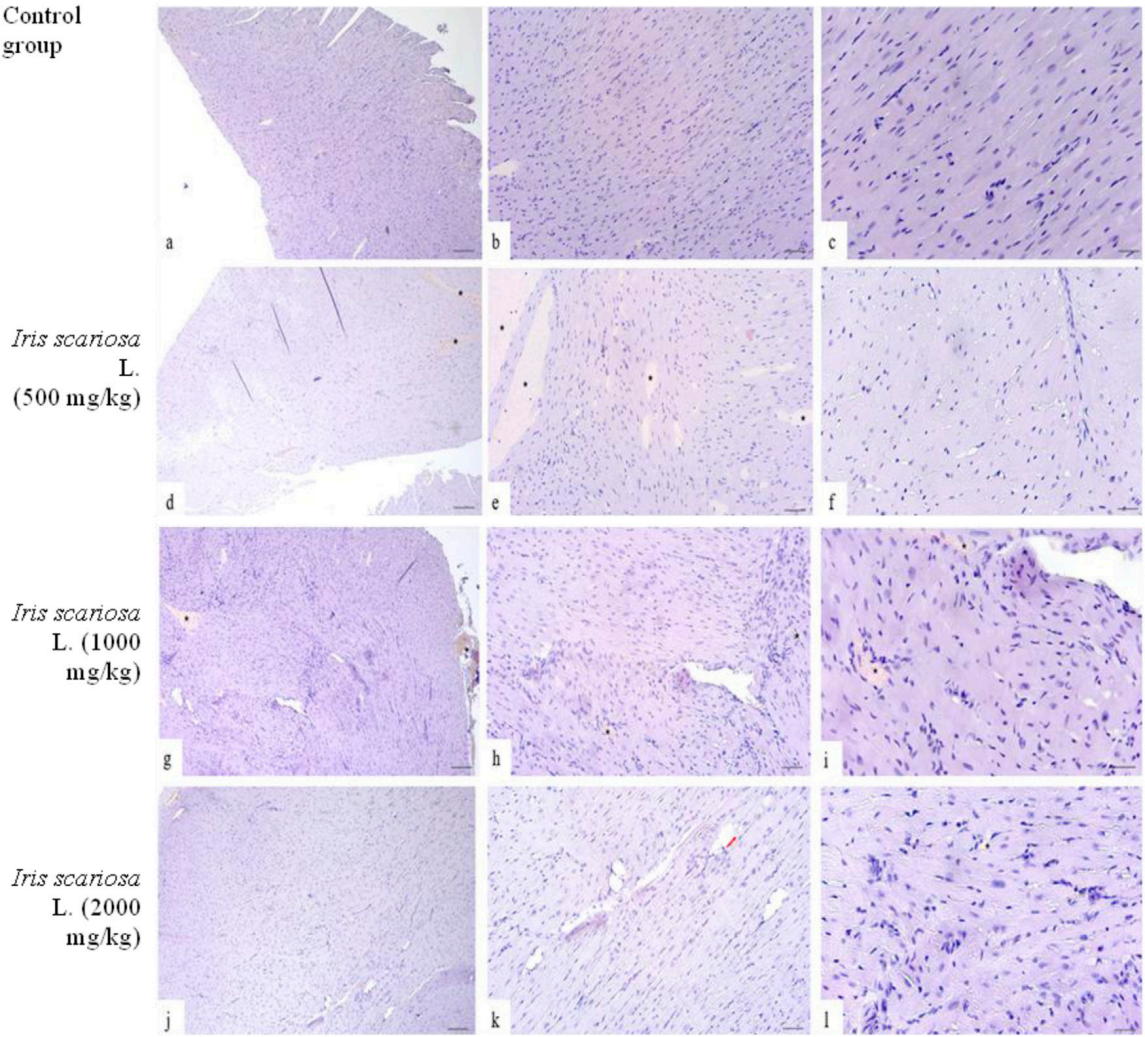
Representative Microscopic Sections of the Heart. H&E Legend: * - venous stasis, red arrow - lipomatosis. **(a–c)** – Control group. The structure of the heart corresponds to the histological norm. **(a)** x40, **(b)** – x100, **(c)** – x200. **(d–f)** – *I. scariosa* L., 500 mg/kg **(d)** - histopathology preserved, cardiomyocytes arranged in an orderly manner, connective tissue represented by a uniform thin interstitium without edema or significant fibrosis. x40. **(e)** - acute venous congestion. x100. **(f)** - cardiomyocytes are arranged in an orderly manner, with clearly defined boundaries, their nuclei are elongated, hyperchromic, centrally located, the structure of myofibrils is preserved, without signs of edema or dystrophic changes. x200. **(g–i)** - *I. scariosa* L., 1,000 mg/kg **(g)** - mild venous congestion. x40. **(h)** - mild dystrophic changes. x100. **(i)** - cardiomyocytes have elongated nuclei, without signs of hypertrophy, degeneration, or necrosis, signs of hydropic dystrophy, and foci of increased eosinophilia of the cell cytoplasm x200. **(j–l)** – *I. scariosa* L., 2000 mg/kg. **(j)** - The myocardium is formed by clearly defined muscle fibers consisting of cardiomyocytes without hypertrophy, pronounced eosinophilia, or transverse striations. x40. **(k)** - Focal lipomatosis. x100. **(l)** - heterogeneity of cardiomyocyte nuclei with areas of focal pyknosis. x200.

In the control group, the structure of the left and right ventricles was histologically normal: histoarchitecture was preserved, cardiomyocytes were arranged in an orderly manner, nuclei were vesicular, hypertrophy and hyper eosinophilia were absent, nuclear atypia, and inflammatory infiltration were absent (Figures 5a–c).

In the group of animals receiving I. scariosa at a dose of 500 mg/kg, half of the animals showed initial vascular stasis and focal interstitial edema (0.5 ± 0.5) (Figures 5d–f), without signs of cardiomyocyte hypertrophy, focal hyper eosinophilia, or changes in cardiomyocyte nuclei.

At a dose of 1000 mg/kg, 6 (75%) animals showed signs of stasis and focal edema of myocardial fibers: of these, 5 (83.3%) cases showed initial changes, and 1 (16.7%) case showed mild venous stasis (0.3 ± 0.5) (Figures 5g–i). Two animals (25%) had isolated perivascular lymphocytes and plasma cells (0.9 ± 0.6 points), and one had mild dystrophic changes in cardiomyocytes, mild intercellular space widening, and interstitial edema.

At a dose of 2000 mg/kg, mild vascular changes were observed in 3 (37.5%) animals and isolated signs of dystrophy and inflammation. In 5 (62.5%) animals, an increase in cardiomyocyte nuclei and the appearance of individual multinucleated cells were noted, and in some places, isolated hyperchromic oval-shaped nuclei were observed (Figures 5j–l).

#### Spleen

Histopathological evaluation of the spleen showed preserved organ architecture in all groups, including the control and experimental groups with the administration of I.scariosa extract at doses of 500 mg/kg, 1000 mg/kg, and 2000 mg/kg. The white pulp contained typical lymphoid follicles with germinative centers of varying intensity, surrounded by mantle and marginal zones, reflecting the functionally active state of the lymphoid tissue (Table 11). The red pulp was clearly differentiated and included erythrocytes, lymphocytes, hemosiderin-containing macrophages, and megakaryocytes. These elements are constant components of normal spleen parenchyma in laboratory animals and indicate preserved phagocytic and hematopoietic activity.

**TABLE 11:** Histopathological evaluation of spleen changes.

Histological criteria	Study groups n = 32								p-value
	Control group (n = 8)		*I. scariosa* L. 500 mg/kg (n = 8)		*I. scariosa* L. 1,000 mg/kg (n = 8)		*I. scariosa* L. 2000 mg/kg (n = 8)		
	M±SD		M±SD		M±SD		M±SD		
Venous congestion	0.0 ± 0.0		0.0 ± 0.0		0.5 ± 0.8		1.3 ± 1.0		p_1_ = 1.00 p_2_ = 0.234 p_3_ = **0.038**
Necrosis	0.0 ± 0.0		0.0 ± 0.0		0.0 ± 0.0		0.0 ± 0.0		p_1_ = 1.00 p_2_ = 1.00 p_3_ = 1.00
^a^ n (%)	0	1	0	1	0	1	0	1	
Hyperplasia/hypoplasia of lymphoid follicles	8 (100%)	0 (0%)	8 (100%)	0 (0%)	8 (100%)	0 (0%)	7 (87.5%)	1 (12.5%)	p_1_ = 1.00 p_2_ = 1.00 p_3_ = 0.302
Atypical forms or pathological enlargement of megakaryocytes	8 (100%)	0 (0%)	8 (100%)	0 (0%)	8 (100%)	0 (0%)	8 (100%)	0 (0%)	p_1_ = 1.00 p_2_ = 1.00 p_3_ = 1.00

^a^ «0» – absence, «1» – presence.

p_1_ < 0.05 – statistically significant differences between the *Iris scariosa* L. group (500 mg/kg) and the control group.

p_2_ < 0.05 – statistically significant differences between the *Iris scariosa* L. group (1,000 mg/kg) and the control group.

p_3_ < 0.05 – statistically significant differences between the *Iris scariosa* L. group (2000 mg/kg) and the control group.

Statistically significant values (p < 0.05) are highlighted in bold.

**FIGURE 6:**
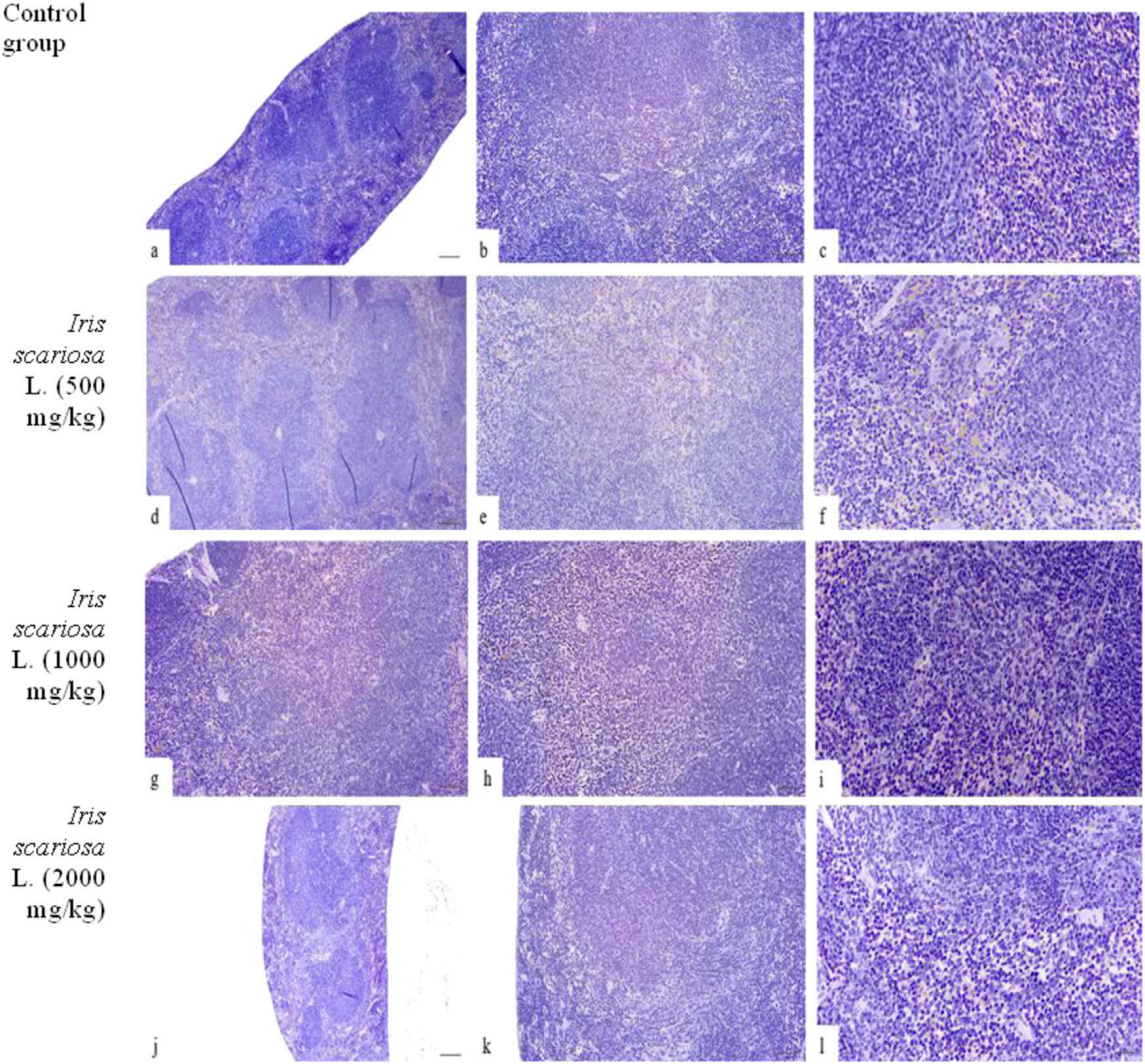
Representative microscopic sections of the spleen. H&E **(a–c)** – Control group. The structure of the spleen corresponds to the histological norm. **(a)** - x40, **(b)** - x100, **(c)** - x200. **(d–f)** – *I. scariosa* L., 500 mg/kg. The histological pattern is preserved. The white pulp consists of lymphoid tissue near the arterioles in the form of spleen follicles. The follicles have germinal centers and a narrow mantle zone at the periphery, beyond which is a wider marginal zone. In addition to a pool of erythrocytes and lymphocytes, the red pulp contains a number of hemosiderin-containing macrophages and megakaryocytes. **(d)** - x40, **(e)** - x100, **(f)** - x200. **(g–i)** – *I. scariosa* L., 1,000 mg/kg. The morphology of the spleen is preserved. The white pulp is represented by numerous lymphoid follicles with germinative centers. The follicles are irregularly distributed, and the boundaries between the white and red pulp are blurred. The trabeculae are poorly visualized, and the vascular structures are not dilated. **(g)** - x40, **(h)** - x100, **(i)** - x200. **(j–l)** – *I. scariosa* L., 2000 mg/kg. The histological picture corresponds to a normal spleen with a clearly formed white pulp and moderate phagocytic activity of the red pulp. **(j)** - x40, **(k)** - x100, **(l)** - x200.

In all dose groups of *I. scariosa* (including the maximum dose of 2000 mg/kg), no necrosis, inflammatory infiltration, or fibrosis was detected, the vessels were not dilated, there was no hemorrhage, and the structure of the trabeculae and capsule was intact (Figure 6). In some cases, when 1000 and 2000 mg/kg were administered, moderate venous congestion was observed without signs of inflammation or dystrophy.

#### Pancreas

**FIGURE 7:**
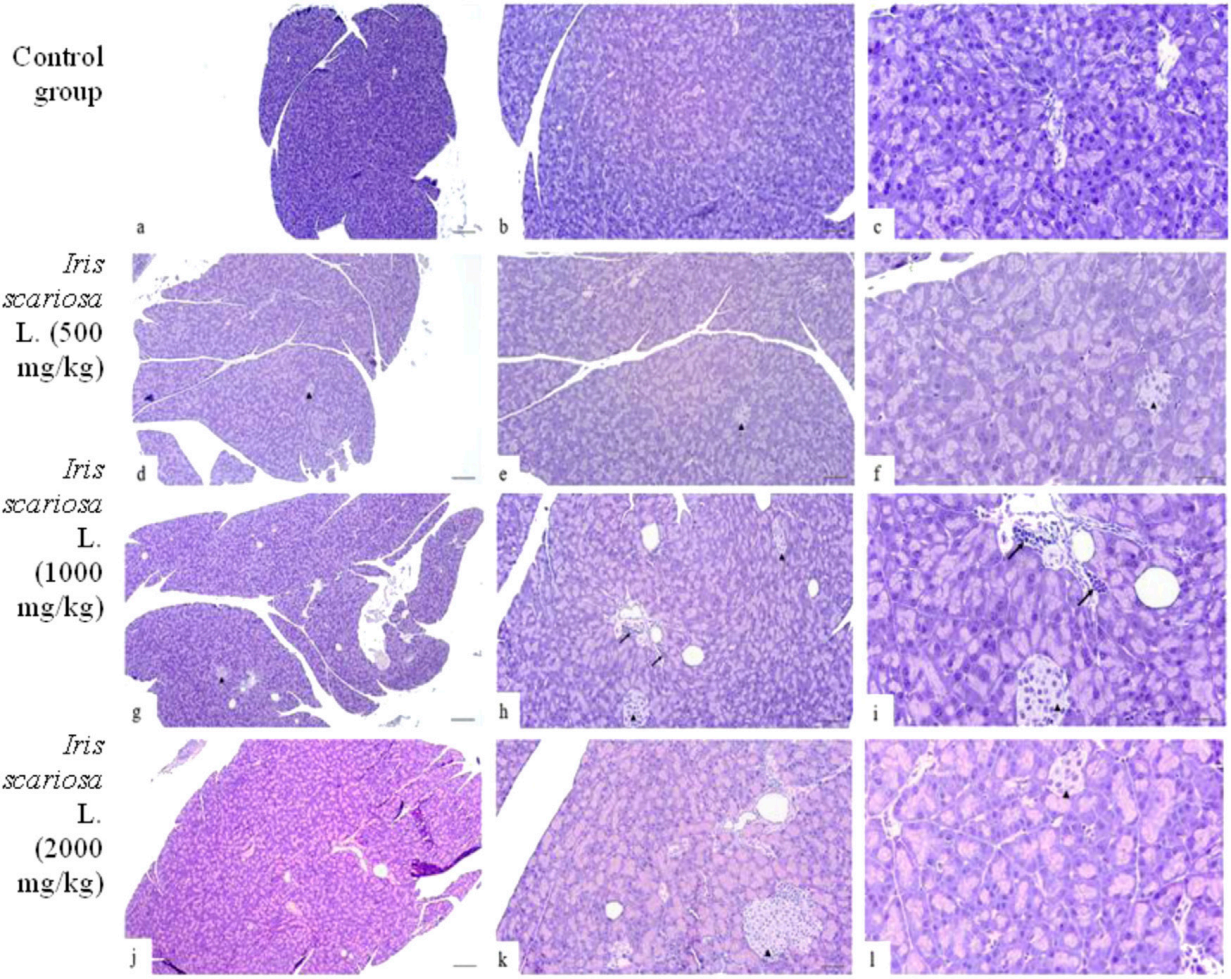
Representative microscopic sections of the pancreas. H&E Legend: black arrows–lymphohistiocytic infiltration, ▲ – islets of Langerhans. **(a–c)** – Control group. The structure of the pancreas corresponds to the histological norm. **(a)** - x40, **(b)** - x100, **(c)** - x200. **(d–f)** - *I. scariosa* L., 500 mg/kg **(d)** - The histoarchitecture of the pancreas is preserved, the cells are basophilic. Thin connective tissue septa containing vessels and ducts are visible between the acini. x40. **(e)** acinar cells are preserved, varying intensity of staining and initial dystrophic changes are noted. x100. **(f)** islets of Langerhans are represented by light cells with clear boundaries. In the acinar cells, there is insignificant variability in the intensity of cytoplasm staining. x200. **(g–i)** - *I. scariosa* L., 1,000 mg/kg **(g)** - The exocrine part of the pancreas is predominantly represented. The acinar structure is preserved, the cells are densely arranged, have basophilic cytoplasm and central/basal nuclei. The vessels are full of blood. x40. **(h)** - scattered perivascular lymphoplasmacytic infiltration. x100. **(i)** - cytoplasm is moderately basophilic, nuclei have smooth contours, without signs of pyknosis or karyorrhexis. Interacinar connective tissue and vascular elements correspond to the histological norm. The islets of Langerhans are clearly visualized, without signs of degeneration or cell density disturbance. Single macrophages and lymphocytes are noted in the stroma. x200. **(j–l)** - *I. scariosa* L., 2000 mg/kg. **(j)** - the histological pattern corresponds to the histological norm. x40. **(k)** - the morphological pattern is preserved, there is variability in the size of the islets of Langerhans and variability in the intensity of cytoplasm staining. x100. **(l)** - Pancreatic tissue is represented by well-organized acinar cells with basophilic cytoplasm and distinct nuclei. There is slight variability in cytoplasm staining. Islets of Langerhans (arrowheads) are preserved and represented by light cells. x200.

A comparative histopathological assessment of pancreatic damage in mice in the study groups is presented in Table 12. In the control group, the histopathological pattern of the pancreas corresponded to the histological norm (Figures 7a–c).

**TABLE 12:** Histopathological evaluation of pancreatic changes.

Histological criteria	Study groups n = 32								p-value
	Control group (n = 8)		*I. scariosa* L. 500 mg/kg (n = 8)		*I. scariosa* L. 1,000 mg/kg (n = 8)		*I. scariosa* L. 2000 mg/kg (n = 8)		
	M±SD		M±SD		M±SD		M±SD		
Cellular infiltrate	0.0 ± 0.0		0.0 ± 0.0		0.8 ± 0.9		0.9 ± 0.6		p_1_ = 1.00 p_2_ = 0.105 p_3_ = **0.010**
Necrosis	0.0 ± 0.0		0.0 ± 0.0		0.0 ± 0.0		0.0 ± 0.0		p_1_ = 1.00 p_2_ = 1.00 p_3_ = 1.00
Venous congestion	0.0 ± 0.0		0.0 ± 0.0		0.4 ± 0.5		0.3 ± 0.5		p_1_ = 1.00 p_2_ = 0.234 p_3_ = 0.442
^a^ n (%)	0	1	0	1	0	1	0	1	
Hyperplasia of the islets of Langerhans	8 (100%)	0 (0%)	8 (100%)	0 (0%)	8 (100%)	0 (0%)	7 (87.5%)	1 (12.5%)	p_1_ = 1.00 p_2_ = 1.00 p_3_ = 0.302
Atrophy of the islets of Langerhans	8 (100%)	0 (0%)	8 (100%)	0 (0%)	8 (100%)	0 (0%)	8 (100%)	0 (0%)	p_1_ = 1.00 p_2_ = 1.00 p_3_ = 1.00
Edema of the interlobular stroma	8 (100%)	0 (0%)	8 (100%)	0 (0%)	8 (100%)	0 (0%)	8 (100%)	0 (0%)	p_1_ = 1.00 p_2_ = 1.00 p_3_ = 1.00
Cell vacuolization	8 (100%)	0 (0%)	8 (100%)	0 (0%)	8 (100%)	0 (0%)	6 (75%)	2 (25%)	p_1_ = 1.00 p_2_ = 1.00 p_3_ = 0.450
Karyopyknosis and karyomegaly	8 (100%)	0 (0%)	8 (100%)	0 (0%)	8 (100%)	0 (0%)	7 (87.5%)	1 (12.5%)	p_1_ = 1.00 p_2_ = 1.00 p_3_ = 0.302
Apoptosis	8 (100%)	0 (0%)	8 (100%)	0 (0%)	8 (100%)	0 (0%)	8 (100%)	0 (0%)	p_1_ = 1.00 p_2_ = 1.00 p_3_ = 1.00

^a^ – «0» – absence, «1» – presence.

p_1_ < 0.05 – statistically significant differences between the *Iris scariosa* L. group (500 mg/kg) and the control group.

p_2_ < 0.05 – statistically significant differences between the *Iris scariosa* L. group (1,000 mg/kg) and the control group.

p_3_ < 0.05 – statistically significant differences between the *Iris scariosa* L. group (2000 mg/kg) and the control group.

Statistically significant values (p < 0.05) are highlighted in bold.

In the group of animals receiving *I. scariosa* at a dose of 500 mg/kg, the overall histoarchitecture of the tissue was preserved in all animals: only slight variability in the intensity of cytoplasmic staining was noted, reflecting initial dystrophic changes. The islets of Langerhans were intact, with no cellular infiltration (Figures 7d–f).

At a dose of 1000 mg/kg, the acinar structure was preserved, with isolated macrophages and lymphocytes noted mainly in the perivascular areas (0.8 ± 0.9) (Figures 7g–i).

At a dose of 2000 mg/kg, minimal vascular changes were detected, including isolated dilated blood vessels and isolated inflammatory cells in the stroma (0.9 ± 0.6). In one case (12.5%), hyperplasia of the islets of Langerhans, karyopyknosis, and karyomegaly were observed, and in two cases (25%), isolated vacuoles in acinar cells were observed (Figures 7j–l).

## Discussion

The results of the assessment of the clinical condition, behavioral reactions, and body weight of the animals during the 14-day observation period after a single intragastric administration of *I. scariosa* L. extract demonstrate its good tolerability. The administration of the test substance was not accompanied by mortality or pronounced clinical manifestations of toxicity. All animals maintained normal motor activity, reflex responses, and appearance, indicating no effect on the central nervous system and general somatic condition.

Analysis of body weight dynamics revealed a stable positive trend in all experimental groups. A slight slowdown in body weight gain in animals receiving *I. scariosa* L. at a dose of 500 mg/kg (n = 8) was not statistically significant. At doses of 1,000 and 2000 mg/kg (n = 8), body weight remained at the level of the control group or slightly exceeded it, which indicates the absence of catabolic effects and, possibly, points to the maintenance of metabolic homeostasis. The absence of significant differences compared to the control (p > 0.05) confirms the absence of adverse effects of the extract on growth parameters.

Data on food and water consumption provide additional confirmation of metabolic stability. At all doses, no significant decrease in consumption was observed; on the contrary, at 1,000 and 2000 mg/kg, there was a tendency toward a moderate increase, especially in the later stages of observation. This may indirectly indicate a slight activating or diuretic effect of the extract at high doses, but the differences were not statistically significant.

The data obtained indicate low acute toxicity of *I. scariosa* L. extract after a single administration, good tolerability, and no signs of behavioral, metabolic, or somatic disorders even at the maximum dose of 2000 mg/kg.

Hematological parameters play a key role in assessing toxicity, reflecting the state of the hematopoietic system and the overall physiological balance of the body. In this study, a single intragastric administration of *I. scariosa* L. extract at doses up to 2000 mg/kg did not cause statistically significant changes in most hematological parameters compared to the control group, indicating its potential safety with regard to the hematopoietic system.

At doses of 500 and 1,000 mg/kg, all key parameters (hemoglobin, erythrocytes, leukocytes, hematocrit, platelets, mean corpuscular volume, etc.) remained within the physiological norm. Although there was a tendency toward an increase in hemoglobin and red blood cell count in the 500 mg/kg group, the differences were not statistically significant (p > 0.05), which may reflect individual biological variations.

Biochemical analysis showed that liver and kidney function did not appear to be impaired by the extract. Total bilirubin levels in the experimental groups did not exceed physiological values, and a moderate increase at a dose of 2000 mg/kg (2.8 ± 0.5 μmol/L vs. 2.4 ± 0.2 μmol/L in the control) was not statistically significant. Similarly, the activity of the liver enzymes ALT and AST showed a tendency to increase at high doses, but the indicators remained within the reference range and did not reach a significant level (p = 0.08 and p = 0.07, respectively). Thus, the data obtained indicate that *I. scariosa* L. extract has no significant toxic effects on the hematopoietic and hepatorenal systems at the doses studied.

No statistically significant differences in toxicity indicators and biochemical parameters were observed between males and females (p > 0.05).

Histological analysis showed that the myocardium, spleen, and pancreas retained their normal morphological organization when I.scariosa extract was administered at all doses studied. The myocardium showed preserved architecture; moderate hyperemia, interstitial edema, and isolated multinucleated cardiomyocytes were episodic and reversible. In the spleen, the structure of the white and red pulp remained unchanged. In the pancreas, the acinar and islet structures were preserved; at 1000–2000 mg/kg, mild perivascular infiltration, minimal cytoplasmic changes, and rare cases of vacuolization or hyperplasia of the islets of Langerhans without signs of apoptosis and fibrosis were observed. In the liver, at 500–1000 mg/kg, only mild focal infiltration and isolated hepatocyte necrosis were observed, accompanied by mild venous stasis, mainly in the central veins; at 2000 mg/kg, these changes were more pronounced but limited in nature, with no signs of fibrosis or massive necrosis. The observed nuclear changes (karyomegaly, hyperchromia) and vacuolization of hepatocytes probably reflect compensatory-adaptive processes rather than a direct hepatotoxic effect.

At the same time, histomorphometric analysis of renal tissue demonstrated a gradual increase in the degree of tubulointerstitial changes depending on the extract dose. In the control group, the structure of the kidneys was normal. At a dose of 500 mg/kg, only isolated areas of mild lymphohistiocytic infiltration and venous stasis (0.6 ± 0.7 and 1.0 ± 0.8 points, respectively) were detected, which is considered a borderline adaptive response without signs of structural damage. At 1000 mg/kg, 87.5% of animals had foci of lymphohistiocytic infiltration, with an average inflammation score of 1.0 ± 0.5; in some animals, there were isolated cell necrosis, epithelial vacuolization, protein cylinder formation, and focal lumen expansion. Taken together, these changes correspond to mild tubulointerstitial damage, manifested mainly at the level of the tubular apparatus and interstitium, without massive necrosis or total disruption of the nephron structure. At 2000 mg/kg, moderate tubulointerstitial damage is observed: marked and moderate foci of infiltration in a significant proportion of animals (mean infiltration score 3.1 ± 0.8), frequent initial and focal necrosis (1.0 ± 0.5), pronounced vacuolization and hyperplasia of the tubular epithelium, formation of protein cylinders, and in 50% of cases, signs of glomerulopathy. Thus, the combination of morphological data and percentage distribution of lesions by group allows us to conclude that I.scariosa extract has dose-dependent nephrotoxicity: at 1000 mg/kg, there is predominantly mild tubulointerstitial damage; at 2000 mg/kg, moderate tubulointerstitial damage with elements of focal necrosis and tubule structure disruption.

Similar mild and reversible morphological changes have previously been described when exposed to naturally occurring phenolic compounds, including p-coumaric acid and catechins, which can exhibit a biphasic effect—antioxidant at low doses and prooxidant at high doses. In particular, p-coumaric acid showed a nephroprotective effect in a cisplatin-induced nephrotoxicity model by inhibiting lipid peroxidation and reducing oxidative stress. Similar results were obtained with the administration of catechin, which prevented tubular cell apoptosis and restored the antioxidant activity of glutathione (Ekinci et al., 2017; Dragoş et al., 2025; Barreiro-Sisto et al., 2024).

Thus, the results obtained allow us to conclude that the morphological changes detected in the kidneys were dose-dependent but reversible, without being accompanied by severe necrotic or destructive processes. They are probably associated with metabolic adaptation to the high content of phenolic compounds in the *I. scariosa* extract, which have both protective and stress-inducing properties when the threshold dose is exceeded. This is consistent with the concept of the hormetic (biphasic) effect of natural antioxidants, in which low doses have a cytoprotective effect, while high doses have a weak cytotoxic effect.

The results of the study demonstrate that *I. scariosa* extract administered intragastrically at doses of 500 mg/kg, 1000 mg/kg, and 2000 mg/kg does not cause severe histopathological changes (Table 13) in the liver, heart, spleen, and pancreas. Moderate morphological changes at high doses are observed in the kidneys, which requires further study of renal safety with repeated administration or chronic use.

**TABLE 13:** Histopathological assessment of prolonged dystrophic (metabolic) and vasculopathic (Ischemic) patterns of damage/reaction in tissues.

Organs	Control group (n = 8)	*I. scariosa* L. 500 mg/kg (n = 8)	*I. scariosa* L. 1,000 mg/kg (n = 8)	*I. scariosa* L. 2000 mg/kg (n = 8)
Kidneys				
Liver				
Heart				
Spleen				
Pancreas				

The histopathological pattern of internal organ injury was determined individually for each tissue, with the primary pattern defined by the criterion representing the main manifestation of vasculopathic cellular injury.

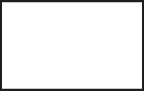
- absent.

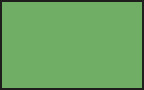
- mild.

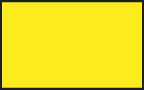
- moderate.

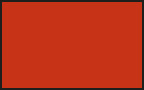
- severe.

This study has a number of limitations. In particular, only acute oral toxicity was assessed for 14 days after a single administration of the extract, and therefore it does not reflect potential delayed or cumulative effects arising from prolonged or repeated use of the *I. scariosa* extract. In addition, subsequent studies should expand the toxicological assessment to include analysis of additional physiological systems and key endpoints, such as neurotoxicity and reproductive toxicity.

## Conclusion

The results of a comprehensive study show that the aqueous extract of *I. scariosa* did not cause acute toxicity after a single intragastric administration at doses of up to 500 mg/kg, 1000 mg/kg, and 2000 mg/kg. During the 14-day observation period, no changes in body weight or deviations in biochemical and hematological blood parameters were observed in the animals, with the exception of a slight increase in urea and transaminase levels at high doses. Histologically, the liver, heart, spleen, and pancreas in all groups retained normal architecture; at high doses, there were mild signs of hyperemia, interstitial edema, and karyomegaly without signs of destruction or inflammatory infiltration. The most pronounced changes were found in the kidneys: at doses of 2000 mg/kg, statistically significant vascular and inflammatory reactions of moderate severity were observed without signs of fibrosis or massive necrosis.

These results indicate that *I. scariosa* rhizome extract is safe for use at the tested doses, while the dose-dependent sensitivity of the kidneys indicates the need for further studies of chronic and subchronic toxicity.

## Data Availability

The original contributions presented in the study are included in the article/supplementary material, further inquiries can be directed to the corresponding author.

## References

[B38] Aitkenova A. A. Ishmuratova M. Yu. Atazhanova G. A. Badekova K.Zh. Tazhina A. M. Sydykova A. Zh. (2025a). Study of the anatomical and morphological structure of the raw material of Iris scariosa L., growing in the territory of Сentral Kazakhstan. Med. Ecol. (3), 130–135. 10.59598/ME-2305-6053-2025-116-3-130-135

[B1] Aitkenova A. A. Ishmuratova M. Y. Atazhanova G. A. Badekova K. Z. Tazhina A. M. Ainur S. Z. (2025b). Histochemical analysis of raw materials of Iris scariosa L. growing in Kazakhstan. Res. J. Pharm. Technol. 18 (5), 2244–2248. 10.52711/0974-360X.2025.00321

[B2] Alekseeva N. B. Petrova T. S. Sidorova E. V. Ivanov V. A. Kuznetsova A. N. (2023). The genus Iris (Iridaceae) in Russia: component composition, biological activity and use in traditional medicine. Plant Resour. 59 (1), 3–29. 10.31857/S003399462301003X

[B3] Amin H. I. M. Farag M. A. Hassan S. M. Abdelkareem M. H. (2017). Chemical composition and antifungal activity of essential oils from Iris persica. Nat. Prod. Commun. 12 (3), 441–444. Available online at: https://pubmed.ncbi.nlm.nih.gov/30549906/ 30549906

[B4] Atazhanova G. A. Badekova K.Zh. Ivasenko S. A. Kacergius T. Levaya Ya.K. Kurmantaeva G. K. (2022). Influence of essential oils on the formation of Streptococcus mutans biofilms. Res. J. Pharm. Technol. 15 (11), 4959–4966. 10.52711/0974-360X.2022.00834

[B40] Bakhoda Mohammadreza Kheiripour N. Ghatre-Samani K. Akhavan Taheri M. Najarzadeh F. Haghighat Lari M. M. (2025). Ameliorating the toxicity induced by L-arginine through iris germanica methanolic extract in an experimental model of acute pancreatitis. Arch. Physiol. Biochem., 1–17. 10.1080/13813455.2025.2541699 40748661

[B41] Barreiro-Sisto U Fernández-Fariña S. González-Noya A. M. Pedrido R. Maneiro M. (2024). Enemies or Allies? Hormetic and Apparent Non-Dose-Dependent Effects of Natural Bioactive Antioxidants in the Treatment of Inflammation. Int. J. Mol. Sci. 25 (3), 1892. 10.3390/ijms25031892 38339170 PMC10855620

[B6] Belozertseva I. Petrova A. N. Sokolov V. I. Ivanov S. V. (2015). Morphine-induced straub reaction: effects of serotonergic compounds. J. Neurol. Psychiatry 4 (2), 73–79. 10.17116/JNEVRO20151154273-79

[B7] Bogdanov A. V. Morozova I. E. Petrov S. V. Kuznetsova N. V. Ivanov A. V. Dobrynin A. B. (2022). Isatin-3-acylhydrazones with enhanced lipophilicity: synthesis, antimicrobial activity evaluation and the influence on hemostasis system. Chem. Biodivers. 19 (2), e202100496. 10.1002/cbdv.202100496 34958705

[B42] Chen F. Zhang Y. Liu J. Li W. Mao J. (2024). P-coumaric acid: Advances in pharmacological research based on oxidative stress. Curr. Top. Med. Chem. 24 (5), 416–436. 10.2174/0115680266276823231230183519 38279744

[B8] Chikhi I. Bensouilah M. Boulekbache-Makhlouf L. Madani K. (2012). Free radical scavenging and antibacterial activity of essential oil and solvent extracts of Iris planifolia (Mill). J. Med. Plants Res. 6 (10), 1961–1968. 10.5897/JMPR11.1668

[B9] Council of Europe (1986). European convention for the protection of vertebrate animals used for experimental and other scientific purposes. Strasbourg: Council of Europe. Available online at: https://rm.coe.int/168007a67b.

[B45] Dragoş D. Enache I. I. Manea M. M. (2025). Oxidative stress and nutritional antioxidants in renal diseases: A narrative review. Antioxidants 14 (7), 757. 10.3390/antiox14070757 40722862 PMC12291686

[B46] Ekinci F. N. Albayrak M. Çalik M Bayir Y. Gülçin İ (2017). The Protective Effects of p-Coumaric Acid on Acute Liver and Kidney Damages Induced by Cisplatin. Biomedicines 5 (2), 18. 10.3390/biomedicines5020018 28536361 PMC5489804

[B47] Fernandes F. H. Salgado H. R. (2016). Gallic acid: Review of the methods of determination and quantification. Crit. Rev. Anal. Chem. 46 (3), 257–265. 10.1080/10408347.2015.1095069 26440222

[B10] Flora of Kazakhstan (1958). Almaty: publishing house of the academy of sciences of the Kazakh SSR. 233–247.

[B11] Ghasemi G. Mousavi Z. Khodadadi B. (2025). Rapid post-harvest processing to enhance thirones in Iris germanica L. J. Agric. Food Res. 20, 101748. 10.1016/j.jafr.2024.101748

[B12] Hoang L. Nguyen T. Le H. Kronusová O. Švarcová V. Řehořová K. (2020). Phytochemical composition and *in vitro* biological activity of Iris spp. (Iridaceae): a new source of bioactive constituents for the inhibition of oral bacterial biofilms. Antibiotics 9 (7), 403. 10.3390/antibiotics9070403 32664528 PMC7399867

[B13] Itzhanova Kh.I. Remetova N. S. Zakareyeva A. K. Abdrakhmanova G. M. Murzaliyeva G. T. Orazbayeva P. Z. (2024). Technology of application dosage form with extract of *Xanthium strumarium* L. for use in dental practice. Res. J. Pharm. Technol. 17 (11), 5561–5566. 10.52711/0974-360X.2024.00849

[B48] Ivasenko S.A. Shakarimova K.K. Bokayeva A.B. Marchenko A.B. Lavrinenko A.V. Kolesnichenko S.I. (2021). Study of the phenolic compounds of the dry extract of Thymus crebrifolius using a combined HPLC–UV and HPLC-ESI-MS/MS method. B. Karag. Univ. Chem. 102 (2), 18–23. 10.31489/2021Ch2/18-23

[B14] Jegnie M. Olumide F. Adebayo T. (2023). Acute and subacute toxicity of Justicia schimperiana leaf extract in rats. J. Exp. Pharmacol. 15, 467–483. 10.2147/JEP.S441273 38026231 PMC10664716

[B49] Jianv L. Xin X. Qixing Z. (2018). Phytoremediation of contaminated soils using ornamental plants. Environ. Rev. 26 (1), 43–54. 10.1139/er-2017-0022

[B15] Keenan C. M. Smith J. P. Roberts L. A. (2015). International harmonization of nomenclature and diagnostic criteria (INHAND): progress and plans. Toxicol. Pathol. 43 (5), 730–732. 10.1177/0192623314560031 25530274

[B16] Khabriev R. U. (2005). Guide to experimental (preclinical) studies of new pharmacological substances. 2nd ed. (Moscow: Meditsina), 832.

[B17] Khatib S. Al-Dhabi N. A. Arasu M. V. Park C. H. (2022). Exploring the use of Iris species: antioxidant properties, phytochemistry, medicinal and industrial applications. Antioxidants 11, 3526. 10.3390/antiox11030526 PMC8944787 35326175

[B51] Khokhlova N. A. Derkach N. V. Zatilnikova O. A. Kovalev V. N. Volkova V. A. (2012). Pharmacological vaccination of Iris Pseudocarus. Ukr. biopharm. j. 1-2, 42–45.

[B18] Lee K. H. Lim T. G. Tan C. (2008). Euphorbia hirta water extracts and cartilage degeneration in rats. Malays J. Pathol. 30, 95–102. Available online at: https://pubmed.ncbi.nlm.nih.gov/19291918/ 19291918

[B19] Levaya Y. Atazhanova G. A. Gabe V. Badekova K. (2025). A review of botany, phytochemistry, and biological activities of eight Salvia species widespread in Kazakhstan. Molecules 30 (5), 1142. 10.3390/molecules30051142 40076365 PMC11901606

[B37] Machalska A. Skalicka-Woźniak K. Widelski J. Głowniak K. Purevsuren G. Oyun Z. (2008). Screening for phenolic acids in five species of Iris collected in Mongolia. Acta Chromatogr. 20, 259–267. 10.1556/AChrom.20.2008.2.10

[B20] Maru S. Belemkar S. (2025). Acute and subacute oral toxicity study of a herbal formulation containing *Asparagus racemosus*, *Tinospora cordifolia*, and *Trigonella foenum-graceum* in mice. J. Toxicol. 2025, 8221552. 10.1155/jt/8221552 39974656 PMC11839261

[B39] Mocan A. Zengin G. Mollica A. Uysal A. Gunes E. Crişan G. (2020). Biological effects and chemical characterization of Iris schachtii Markgr. extracts: A new source of bioactive constituents. Food Chem. Toxicol. 112, 448–457. 10.1016/j.fct.2017.08.004 28797651

[B21] Morozova J. E. Bogdanov A. V. Petrov S. V. (2022). Calix[4]resorcinarene carboxybetaines and esters: *in vitro* toxicity, anti-platelet, anticoagulant activity and BSA binding. Int. J. Mol. Sci. 23, 15298. 10.3390/ijms232315298 36499625 PMC9740030

[B22] Mykhailenko O. Petrenko S. Ivanova T. Bezruk I. Myhal A. Petrikaitė V. (2020). Qualitative and quantitative analysis of Ukrainian Iris species: a fresh look on their antioxidant content and biological activities. Molecules 25 (19), 4588. 10.3390/molecules25194588 33050063 PMC7582944

[B23] Niazi A. A. Khan M. I. Ahmed S. (2021). Eugenol improves liver damage from fructose-rich diet. Adv. Biomed. Res. 10, 42. 10.4103/abr.abr_237_20 35071110 PMC8744418

[B24] OECD (2025). Test No. 407: repeated dose 28-day oral toxicity study in rodents. OECD Guidel. Test. Chem. 10.1787/9789264070684-en

[B25] Okba M. M. Hassan H. M. El-Sayed W. Shehabeldine A. M. El-Sherei M. M. Khaleel A. E. (2022). UPLC-ESI-MS/MS profiling of the underground parts of common Iris species in relation to their anti-virulence activities against *Staphylococcus aureus* . J. Ethnopharmacol. 282, 114658. 10.1016/j.jep.2021.114658 34555449

[B26] Omarova B. A. Tleugabylov K. T. Smagulov M. K. Abekova А. О. Ishmuratova M. Y. Petrova T. N. (2024). Biological effects and phytochemical study of the underground part of iris scariosa Willd. ex Link extract: a new source of bioactive constituents. Fitoterapia 175, 105920. 10.1016/j.fitote.2024.105920 38531480

[B43] Organisation for Economic Co-operation and Development (OECD) (2001). OECD Test Guideline 425: Acute Oral Toxicity—Up-and-Down Procedure. Paris, France: OECD Publishing.

[B27] Plant of the World Online (2025). Genus Iris. Available online at: https://powo.science.kew.org/taxon/urn:lsid:ipni.org:names:326330-2.

[B44] Peng H. Y. Zhang X. H. Xu J. Z. (2016). Apigenin-7-O-β-D-glycoside isolation from the highly copper-tolerant plant Elsholtzia splendens. J. Zhejiang Univ. Sci. B 17 (6), 447–454. 10.1631/jzus.B1500242 27256678 PMC4913793

[B28] Sall I. M. Haşaş A. D. Malek A. Vodnar D. C. Aziez M. Semzenisi E. (2025). Acute and subacute oral toxicity assessment of Kinkeliba (Combretum micranthum G. Don) ethanolic extract in BALB/c mice. Plants 14, 1776. 10.3390/plants14121776 40573764 PMC12196914

[B29] Snyder J. M. Brown T. Wilson A. (2016). Cause-of-death analysis in rodent aging studies. Vet. Pathol. 53 (2), 233–243. 10.1177/0300985815610391 26508696

[B30] Somarathna T. Perera H. Gunasekara R. (2021). Toxicity profiles of Alpinia malaccensis extract in rat and cell models. J. Toxicol. 2021, 9578474. 10.1155/2021/9578474 33531897 PMC7834801

[B31] Tikhomirova L. I. Petrova N. V. Morozova J. E. (2015). Pharmacological and biochemical rationale for practical use of some Iris L. species (review). Chem. Plant Raw Mater 3, 25–34. Available online at: https://journal.asu.ru/cw/article/view/837.

[B32] Tine Y. Diallo M. Sylla A. Diallo A. Ndiaye B. Ndoye I. (2024). Combretum micranthum G. Don (Combretaceae): a review on traditional uses, phytochemistry, pharmacology and toxicology. Chem. Biodivers. 21 (5), e202301606. 10.1002/cbdv.202301606 38353648

[B33] Veteläinen R. L. Heikkilä P. Koskinen H. van Vliet A. van Gulik T. M. (2006). Hepatobiliary function assessed by 99mTc-mebrofenin cholescintigraphy in the evaluation of severity of steatosis in a rat model. Eur. J. Nucl. Med. Mol. Imaging 33 (10), 1107–1114. 10.1007/s00259-006-0125-3 16738848

[B34] World Health Organization (2013). WHO traditional medicine strategy: 2014–2023. Geneva: WHO. Available online at: https://iris.who.int/handle/10665/92455.

[B35] Yousefsani B. S. Moradi M. Hosseini M. Jamshidi A. Dadmehr M. (2021). A review on phytochemical and therapeutic potential of Iris germanica. J. Pharm. Pharmacol. 73 (5), 611–625. 10.1093/jpp/rgab008 33772287

[B50] Zakia D. A. Syukri D. Rini R. Anggraini T. Ahmed U. Rahmi Z. J. A. Handra H. (2025). Foundational study on the stability limitations of catechin from Uncaria gambier in aqueous systems: Basis for industrial applications. Asian J. Green Chem. 9 (5), 573–586. 10.48309/AJGC.2025.513983.1716

[B36] Zholdasbayev M. E. Atazhanova G. A. Musozoda S. Poleszak E. (2023). Prunella vulgaris L.: an updated overview of botany, chemical composition, extraction methods, and biological activities. Pharmaceuticals 16, 1106. 10.3390/ph16081106 37631021 PMC10460042

